# Structural mechanisms for cold‐adapted activity of phosphoenolpyruvate carboxykinase

**DOI:** 10.1002/pro.70326

**Published:** 2025-10-16

**Authors:** Matthew J. McLeod, Shauhin Yazdani, Sarah A. E. Barwell, Todd Holyoak

**Affiliations:** ^1^ Department of Biology University of Waterloo Waterloo Ontario Canada; ^2^ Present address: Department of Chemistry University of Cincinnati Cincinnati Ohio USA

**Keywords:** enzyme structure‐function, omega loops, phosphoenolpyruvate carboxykinase, psycrophillic enzymes, thermal adaptation

## Abstract

Temperature is a critical factor in enzyme function, as most enzymes are thermally activated. Across Earth's diverse environments (−20 to 120°C), enzymes have evolved to function optimally at their organism's growth temperature. Thermophilic enzymes must resist denaturation, while psychrophilic enzymes must maintain activity with limited thermal energy. Although principles underlying thermostability are well established, the mechanisms governing kinetic adaptation to temperature remain less understood. To investigate this, we characterized the kinetics and determined a comprehensive series of X‐ray crystal structures of a psychrophilic, GTP‐dependent phosphoenolpyruvate carboxykinase (PEPCK) bound to substrates and non‐reactive mimics of the reaction coordinate. These structures were compared to those of a mesophilic PEPCK. PEPCK is a dynamic enzyme requiring substantial conformational changes during catalysis, particularly ordering of the active site Ω‐loop lid. The psychrophilic enzyme exhibited a reduced catalytic efficiency (*k*_cat_/*K*_M_) and lower optimal temperature (*T*_opt_) relative to its mesophilic counterpart. Structural comparisons revealed substitutions in the Ω‐loop that likely increase the entropic cost of loop ordering and reduce enthalpic stabilization, hindering efficient active site closure. These results provide a mechanistic basis for cold adaptation in enzyme catalysis, linking specific structural features to altered kinetic behavior. Understanding such adaptations not only advances our knowledge of enzyme evolution but also informs protein engineering efforts aimed at designing efficient biocatalysts for industrial applications operating at non‐physiological temperatures.

## 1 INTRODUCTION

Enzymes are dynamic molecular scaffolds capable of adopting various conformational states (Frauenfelder et al., 1991; Karplus & McCammon, 1983). As the enzyme‐substrate complex is formed, the thermodynamics of the enzyme change, favoring productive conformations with active site geometries optimized for chemistry (Koshland, 1958). This connection between conformational sampling and the reaction coordinate links enzyme dynamics with function (Eisenmesser et al., 2005). An enzyme's conformational sampling and underlying free‐energy landscape are highly dependent on its environment (Guerrero et al., 2024). Among environmental parameters, temperature is especially important as most enzymes (unless photoactive) are thermally activated. Functional dynamics and enzyme stability are optimized to the temperature at which the enzyme has evolved (Wolf‐Watz et al., 2004). Life exists across a wide range of thermal niches, where microorganisms have been discovered spanning a temperature range of ~−20 to 121°C, suggesting that there is a wide range of thermal set‐points enzymes can adapt to (Blöchl et al., 1997; Mykytczuk et al., 2013). However, once evolved it is difficult to maintain function outside of a narrow window as evidenced by the observation that psychrophilic organisms cannot live in moderate to high thermal environments and vice‐versa (Marchant et al., 2002; Xu et al., 2003).

As temperature increases, both non‐enzymatic and enzyme‐catalyzed reaction rates increase, albeit with different temperature coefficients (Elias et al., 2014). However, it has been observed that at increased temperature (above *T*_opt_), activity decreases but the enzyme's tertiary structure may be maintained. Although there is little structural data supporting how the enzyme scaffold becomes inactive before unfolding at modestly elevated temperatures, one proposed mechanism is that the entropic penalties for ordering important catalytic residues or loops are increased (McLeod et al., 2025; Roy et al., 2017). At even higher temperatures, the tertiary structure unfolds, resulting in complete loss of function. To counteract thermal denaturation at high temperatures, (hyper)thermophilic enzymes have evolved to have an increased number of stabilizing forces (e.g., salt interactions, H‐bonding, etc.) (Pucci & Rooman, 2017). It has been suggested that increased thermostability rigidifies the scaffold, keeping an enzyme folded at high operational temperatures. Despite the rigidification, the higher thermal energy can be partitioned into the conformational landscape to maintain functional dynamics (Fusco et al., 2022; Wolf‐Watz et al., 2004). In contrast, when thermophilic enzymes are at sub‐optimal temperatures, inactivation is thought to result from insufficient thermal energy required to induce functional conformation changes in the rigid enzyme. Therefore, enzymes must evolve to carefully balance structural stability while allowing sufficient flexibility to promote productive conformational sampling (Low & Somero, 1974). This activity–stability tradeoff has been extensively described (Siddiqui, 2017; Siddiqui & Cavicchioli, 2006). However, there are numerous examples where enzymes may be both highly thermostable and active at low temperatures, suggesting the stabilizing sites are decoupled from functional ones (Gatti‐Lafranconi et al., 2008; Miyazaki et al., 2000; Nguyen et al., 2017). This tradeoff may only be valid on a case‐by‐case basis rather than a requirement of thermal evolution (Miller, 2017; Stark et al., 2022).

The study of enzyme thermal evolution has typically focused on describing residue changes leading to thermal stability (Pucci & Rooman, 2017). Design principles describing functional optimization at different temperatures are less understood. When “universal” rules of thermal adaptation have been offered (e.g., stability–activity tradeoff), with time they become less clear, likely due to an insufficient number of examples. A more fruitful approach to understanding thermal adaptation may be to rationalize the connection between structural changes of functional motifs and measured kinetic differences between evolved variants. To gain insight into how evolution adapts functional dynamics to low temperature, we have collected a set of kinetic measurements and high‐resolution crystal structures of a psychrophilic GTP‐dependent phosphoenolpyruvate carboxykinase (*Polaromonas naphthalenivorans*; *Pn*PEPCK—*T*_growth_ ~ 4–20°C, *T*_opt_ ~ 20°C) (Jeon et al., 2004).

**FIGURE 1:**
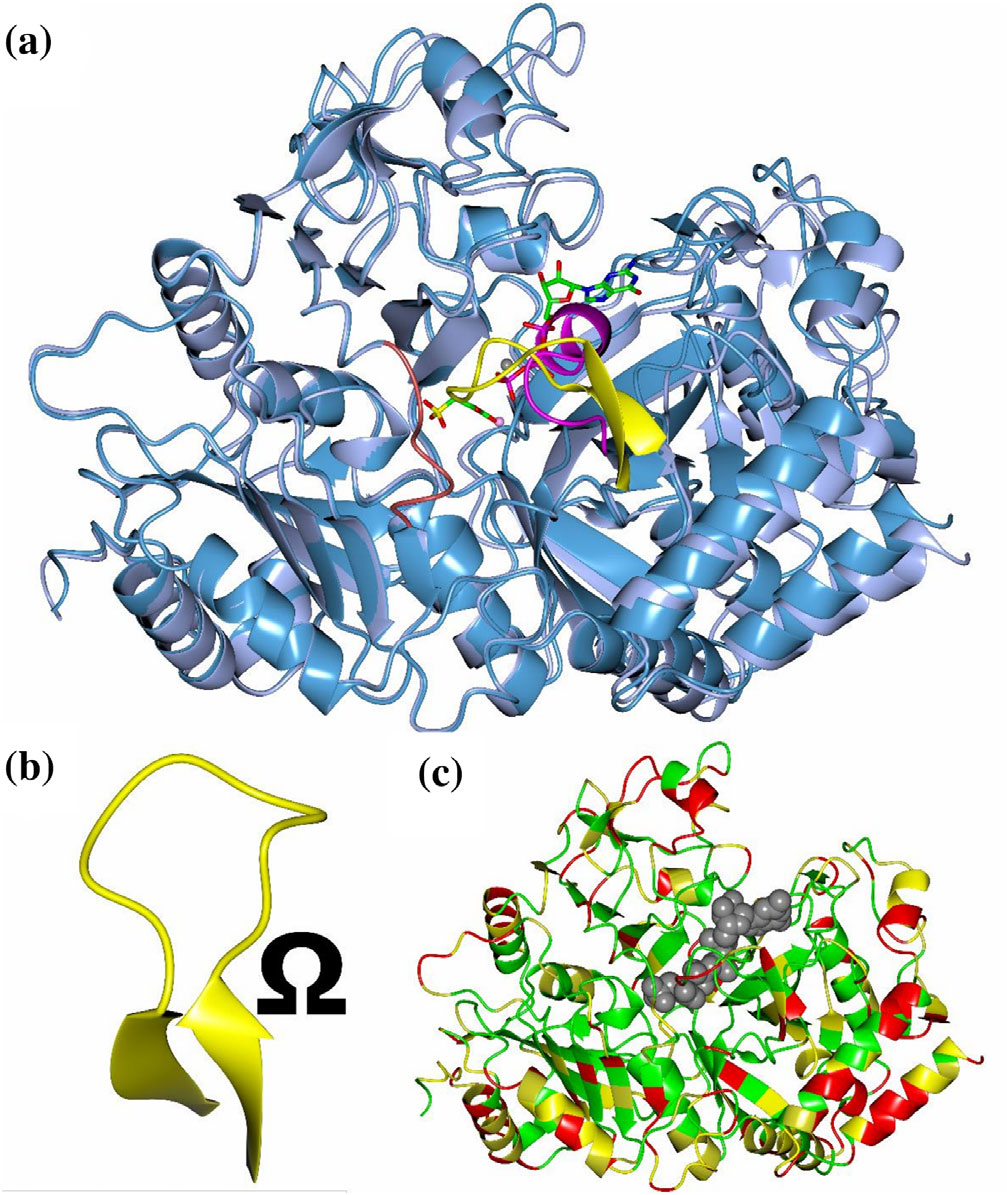
Global conformational change of open‐close transition of *Pn*PEPCK and Ω‐loop structure. (a) Open holo *Pn*PEPCK (pale blue) structure aligned (via N‐terminal domain: residues 1–200) with closed βSP‐GTP complex (dark blue) shows a global structural change where the N‐ and C‐terminal domains rotate towards the active site. Active site loops are shown; R‐loop—firebrick red, P‐loop—magenta, Ω‐loop—yellow. The oxaloacetic acid analog β‐sulfopyruvate (βSP) and phosphoryl donor GTP are colored by atom type; carbon—green, oxygen—red, nitrogen—blue, phosphorous—purple, sulfur—yellow. (b) Ω‐loop structure. The latch (apex) is shown as a loop, while the hinge region is shown as β‐sheet. (c) Sequence conservation between *Pn*PEPCK and rcPEPCK. Green is complete conservation, yellow indicates partial conservation, and red non‐conserved, ligands in the active site are shown as gray spheres.

PEPCK is an important metabolic enzyme that catalyzes the bidirectional reaction between oxaloacetic acid (OAA) and phosphoenolpyruvate (PEP) using a phosphoryl donor (GTP, ATP, or PP_i_, depending on class) (McLeod & Holyoak, 2021). This reaction proceeds (in the “forward” direction) by the decarboxylation of OAA to form an enol‐pyruvate intermediate which is then phosphorylated to create PEP. Years of structure–function studies on the GTP‐dependent, mesophilic rat cytosolic PEPCK (rcPEPCK) have provided insight into the molecular events facilitating the transition from an open to a closed (active) conformation after substrate binding (Carlson & Holyoak, 2009; Holyoak et al., 2006; Sullivan & Holyoak, 2007; Sullivan & Holyoak, 2008). PEPCK has three active site loops: the R‐loop, which coordinates the substrate (OAA, enol‐pyruvate intermediate, or PEP); the P‐loop, which coordinates the nucleotide or PP_i_; and the Ω‐loop, a flexible lid that is disordered when “open” and orders/closes over the active site after the Michaelis complex is formed (Figure 1a) (Sullivan & Holyoak, 2008; Holyoak et al., 2006). Ω‐loops are non‐regular secondary structure elements that are characterized by a narrow beginning and ending (β‐sheet structure; Figure 1b) with an extended medial region resembling the shape of the Greek letter omega (Fetrow, 1995). As PEPCK's Ω‐loop must undergo a disorder‐to‐order transition, and the sampling between these two states is essential for activity (Cui et al., 2017; Johnson & Holyoak, 2010; Johnson & Holyoak, 2012; Johnson et al., 2016), we suspect that evolutionary pressures like temperature will directly change the sequence of the loop and surrounding regions that play a role in the required disorder/order transition. For example, the lid must be disordered (opened) for substrates to bind and must then become ordered (closed) for the reaction to proceed. Closure must be maintained during the two‐step chemical reaction, and premature opening will lead to pyruvate formation by solvent protonation of the enol–pyruvate intermediate (Johnson & Holyoak, 2010; Johnson & Holyoak, 2012). Finally, the lid must reopen to release the product and restart its catalytic cycle. Lid ordering is achieved and maintained by interactions that form at the apex of the loop with the R‐loop, as well as dynamic transitions at the β‐strands that form the beginning and ending structures of the loop as well as their neighboring regions (latch and hinge respectively—*vida infra* Figure 1b) that must undergo specific conformational changes (Sullivan & Holyoak, 2008; Cui et al., 2017).

To understand how thermal pressures have led to the evolution of enzyme activity and dynamics, we compare kinetic measurements and crystal structures of *Pn*PEPCK to their rcPEPCK counterparts previously published. PEPCK was studied here as its rich history allows kinetic parameters to be correlated with functional dynamics. rcPEPCK was used as a mesophilic comparison as it is the best studied GTP‐dependent PEPCK. Although our studies found differences outside the active site and there are a variety of sequence differences between the two enzymes (Figure 1c), we have focused our analysis on detailing the dynamics of the active site loops as they have clearly described roles in activity and can be better correlated with similarities and/or differences in experimentally derived kinetic constants and their temperature dependencies.

## 2 MATERIALS AND METHODS

Lactate dehydrogenase, malate dehydrogenase, and pyruvate kinase for enzyme‐coupled kinetics were purchased from Calzyme. GTP and PEP were purchased from ChemImpex. Phosphoglycolic acid (PGA) and oxalate were purchased from Sigma‐Aldrich. βSP was synthesized in the lab as previously described (Griffith & Weinstein, 1987). TCEP and DTT were purchased from GoldBio. Nickel‐NTA was purchased from UBP‐Bio, while P6‐DG resin was purchased from BioRad. All other materials were purchased at the highest grade available.

### 2.1 Constructs, expression and purification

The codon‐optimized gene for *Polaromonas naphthalenivorans* GTP‐dependent PEPCK (EC: 4.1.1.32, Seq ID: WP_011799537.1) was synthesized and cloned into pESUMO‐Star (Kan) by GenScript. The final expressed protein had a SUMO 6‐His fusion tag on the N‐terminus, but incubation with SUMO protease results in the full‐length protein without any additional amino acids.

Both rc‐ and *Pn*PEPCK were expressed in BL21(DE3) cells. Cultures were first grown in LB media (50 mL, 50 μg/mL kanamycin) overnight. Overnight cultures were centrifuged and inoculated into fresh ZYP‐5052 autoinduction media (1 L, 50 μg/mL kanamycin) and cultured at 20°C for 20 h (Studier, 2005). Cells were harvested by centrifugation and frozen as cell pellets at −80°C until use.

Frozen cell pellets were resuspended in buffer 1 (25 mM HEPES‐OH pH 7.5, 10 mM imidazole, 300 mM NaCl, 10% (w/v) glycerol, 2 mM TCEP) and lysed with a chilled French press at 1000 PSI. Lysed cells were centrifuged at 12,000 × g for 45 min, and the recovered supernatant was incubated with pre‐equilibrated Ni‐NTA resin for 1 h. Resin was washed with buffer 1 until an *A*_280_ < 0.1 and eluted with buffer 2 (25 mM HEPES‐OH pH 7.5, 300 mM imidazole, 2 mM TCEP). Fractions containing protein were concentrated, passed through a P6‐DG column to buffer exchange into buffer 3 (25 mM HEPES‐OH pH 7.5, 2 mM TCEP), and left to incubate with 1 mg SUMO protease (purified as previously described) (Johnson & Holyoak, 2012) overnight at 4°C. Digested protein was added back onto cleaned Ni‐NTA resin pre‐equilibrated in buffer 3 for 1 h. Flowthrough containing protein was collected and buffer exchanged into buffer 4 (25 mM HEPES‐OH pH 7.5, 10 mM DTT) and flash frozen at 20 mg/mL in 30 μL aliquots. Protein concentration was determined using an extinction coefficient (*E*_280,1%_) of 10.9 (rcPEPCK) and 20.5 (*Pn*PEPCK) as determined by ExPasy ProtParam (Gasteiger et al., 2005).

### 2.2 Kinetics

Thermal inactivation experiments were conducted using the standard reaction mixture below. PEPCKs were diluted to working concentrations and incubated in PCR tubes in a thermocycler. Small aliquots were removed at various time points and tested for activity. Activity measurements were normalized to the activity measured prior to thermal incubation.

PEP‐dependent kinetic constants were determined by holding all other substrates constant. For Arrhenius/Eyring kinetics, saturating concentrations of all substrates were used. Kinetic rates were measured by the observance of a decrease in absorbance at 340 nm due to the oxidation of NADH in the coupled enzymatic reaction using a CaryUV100 spectrophotometer and a temperature controller (temperatures verified with thermocouple). Reactions were a total volume of 1 mL and were measured in duplicate, and errors are reported as standard errors of the mean.

The standard reaction mixture to collect data was 100 mM HEPES‐OH pH 7.5, 10 mM DTT, 300 μM NADH, 4 mM MgCl_2_, 1 mM GDP, (4:1 metal: nucleotide ratio), 100 μM MnCl_2_, 10 mM PEP, 50 mM KHCO_3_ (bubbled with dry ice), 10 U MDH, and 2.5 μg of *Pn*PEPCK. PEP was varied to calculate Michaelis–Menten kinetic constants (Equation 1) using SigmaPlot Enzyme Kinetics package. Temperature‐dependent Arrhenius/Eyring constants were determined using the linearized equation (Equation 2a and 2b) using an R‐script.

(1) $$v=\frac{V_{\max,\mathrm{app}}•\left[S\right]}{K_{M,\mathrm{app}}+\left[S\right]}$$

(2a) $$k_{\mathrm{cat}}\left(T\right)=\frac{k_{B}•T}{h}e^{\frac{∆S\left(T\right)}{R}}•e^{\frac{-∆H\left(T\right)}{R•T}}$$

(2b) $$\ln\left(\frac{k_{\mathrm{cat}}\left(T\right)}{T}\right)=\frac{-∆H\left(T\right)}{R}•\frac{1}{T}+\ln\frac{k_{B}}{h}+\frac{∆S\left(T\right)}{R}$$

∆*S*(*T*), ∆*H*(*T*), and *k*_cat_(*T*) denote that these are temperature dependent parameters (McLeod et al., 2025).

### 2.3 Differential scanning fluorimetry

Differential scanning fluorimetry (DSF) was used to determine melting temperatures (*T*_M_). rcPEPCK and *Pn*PEPCK at 21.4 μM were prepared in 25 mM HEPES, pH 7.4, and 1 mM MnCl_2_. SYPRO Orange was added to a 10× final concentration from a 5000× stock. Where indicated, GTP and/or oxalate were each included at a concentration of 214 μM (10:1 ligand:protein ratio). Final reaction volumes were 20 μL per well, and each condition was run in triplicate. Thermal scans were performed on a QuantStudioPro 6 thermocycler. Reactions started at 20°C, ramped at +0.05°C/s to 90°C, were held for 5 s, and then cooled to 20°C at −3°C/s. Fluorescence data were collected every 3 seconds throughout the run. Raw fluorescence traces were processed using QuantStudio Design & Analysis Software (v2.8.0). Melt curve plots, −dRFU/d*T* against temperature, were generated, and *T*_M_ values were determined from the peak minima of the derivative curves (Figures S1 and S2).

### 2.4 Crystallography and structure determination

To form representative complexes, both native substrates and inhibitors mimicking substrates were used (Stiffin et al., 2008). PEP was used to form the binary Mn^2+^‐PEP complex, while beta‐sulfopyruvate (βSP) was used to form a binary complex representing the Mn^2+^‐OAA substrate complex. To form representative ternary complexes, βSP was used to replace OAA (paired with GTP), oxalate was used to mimic the enol‐pyruvate intermediate (paired with GTP), and PGA was used to replace PEP (paired with GDP).

Initial cocrystals were grown with 10 mM of both GTP and oxalate at 4°C through vapor diffusion. Mother liquor conditions were 100 mM TRIS‐Cl pH 8.0, 0.8 M LiCl_2_, 5 mM MgCl_2_, 1 mM MnCl_2_, and 30–40% (w/v) PEG 8000. These crystals were then used for seeding. Seeding experiments were completed as above, but at room temperature and with 0.4 M LiCl_2_, where mixed drops (supplemented with ~10 mM of each substrate/inhibitor‐mimic depending on the complex obtained) were allowed to equilibrate for 1 day. These drops were then seeded with crushed microcrystals and allowed to nucleate. Once grown, crystals were harvested and cryoprotected in the mother liquor condition with an additional 10% (v/v) PEG 400. Crystals were cryocooled by immersion in liquid nitrogen.

The P2_1_2_1_2_1_ holo structure was obtained when the drops were supplemented with 10 mM PGA, despite no evidence of PGA being present. When 10 mM PEP was included during crystallization, two crystal morphologies were present—rods (C222_1_) and prisms (P2_1_2_1_2_1_) both with PEP bound at the active site.

Data on the GTP–oxalate complex was collected at the University of Waterloo's home source rotating copper anode, and data were integrated, scaled, and merged with HKL 2000 (Otwinowski & Minor, 1997). All other data were collected at Cornell High Energy Synchrotron Source (CHESS) at the 7B2 end‐station. Data were integrated and scaled with DIALS (Winter et al., 2018) and merged with AIMLESS (Evans & Murshudov, 2013). Molecular replacement with MOLREP (Vagin & Teplyakov, 1997) using *Mycobacterium tuberculosis* PEPCK was used for initial phases (PDB 4R43) (Machová et al., 2015). After the oxalate‐GTP complex was determined, it was then used as the search model for the other complexes. Refinement and model building were completed using phenix.refine (Afonine et al., 2012) and COOT (data collection and refinement statistics are presented in Tables S3 and S4) (Emsley & Cowtan, 2004). Structures were validated using the MolProbity online server (http://molprobity.biochem.duke.edu/index.php) (Chen et al., 2010).

## 3 RESULTS

### 3.1 Kinetic evaluation of *Pn*PEPCK

**FIGURE 2:**
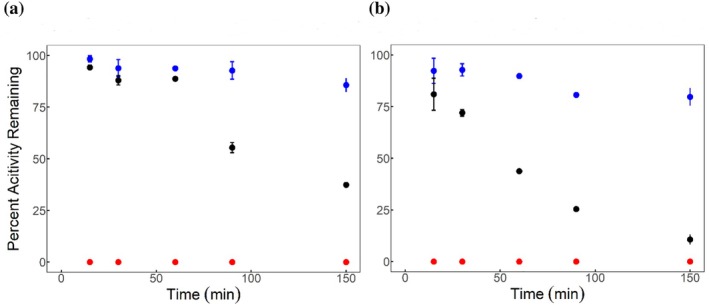
Thermal inactivation of PEPCKs. (a) *Pn*PEPCK inactivation and (b) rcPEPCK inactivation after incubation at 4°C (blue), 37°C (black), and 55°C (red). Data were measured in duplicate and error bars (standard error) may be hidden within the data point.

Thermostability was first evaluated by incubating both mesophilic (rc‐) and psychrophilic (*Pn*‐) PEPCK at 4, 37, and 55°C and checking activity over time to determine the rate of thermal inactivation for each enzyme. At 4°C, both enzymes were stable for the entire time course (150 min) (Figure 2). Surprisingly, at 37°C both enzymes were quite stable, where *Pn*PEPCK inactivated to 50% residual activity at ~114 min, whereas rcPEPCK retained 50% activity at ~49 min. Finally, at 55°C both enzymes were fully inactive at the shortest time point measured (15 min). These assays were complemented with differential scanning fluorimetry measurements to determine thermal melting temperatures. Both rc‐ and *Pn*PEPCK were tested without ligands but with manganese present at 1 mM (holo), with oxalate and manganese, with manganese and GTP, and with manganese, oxalate, and GTP to mimic the ternary complex. rcPEPCK's observed *T*_M_'s were 52.0, 54.4, 61.4, and 64.7°C, while *Pn*PEPCKs were *T*_M_'s were 42.7, 46.3, and 59.5°C for each complex, respectively (Table 1).

**TABLE 1:** Melting temperatures (DSF) of each PEPCK variant and complex.

	rcPEPCK	*Pn*PEPCK	
	*T*_M_ (°C)	*T*_M_ (°C)	∆*T*_M (rc‐*Pn*)_
Mn	52.0	42.7	9.3
Mn‐Oxalate	54.4	46.3	8.1
MnGTP	61.4	59.5	1.9
Mn‐Oxalate‐MnGTP	64.7	61.7	3.0

Second, kinetic analyses of *Pn*PEPCK were carried out at four different temperatures (15, 25, 37, and 45°C) (Table 2 and Figure S3). Similar measurements were carried out for rcPEPCK in previous work (highest temperature sampled was 55°C) (McLeod et al., 2025). Generally, *K*_M_ and *k*_cat_ increased with respect to temperature for both enzymes. At 45°C, *Pn*PEPCK activity decreased while rcPEPCK activity continued to increase, surpassing *Pn*PEPCKs maximal activity. The *K*_M_ value for *Pn*PEPCK at all temperatures was greater than rcPEPCK, and the corresponding *k*_cat_/*K*_M_ value indicated that *Pn*PEPCK was approximately an order of magnitude less efficient at capturing substrate and committing to product formation.

**TABLE 2:** Temperature dependence of kinetic constants—PEP varied; PEP + GDP + CO_2_ ➔ OAA + GTP.^a^

*T* (°C)	Enzyme	*K*_M_ (μM)	*k*_cat_ (s^−1^)	*k*_cat_/*K*_M_ (M^−1^ s^−1^)
15	*Pn*PEPCK	810 ± 87	5.8 ± 0.19	7.2 × 10^3^
	rcPEPCK	120 ± 8.7	6.0 ± 0.01	5.0 × 10^4^
25	*Pn*PEPCK	2500 ± 650	23 ± 1.4	9.2 × 10^3^
	rcPEPCK	200 ± 23	12 ± 0.34	6.5 × 10^4^
37	*Pn*PEPCK	3400 ± 290	92 ± 3.2	2.7 × 10^4^
	rcPEPCK	330 ± 34	71 ± 1.7	2.2 × 10^5^
45	*Pn*PEPCK	5900 ± 1000	47 ± 3.0	8.0 × 10^3^
55	rcPEPCK	830 ± 110	230 ± 7.1	2.8 × 10^5^

^a^ rcPEPCK data was previously published (McLeod et al., 2025).

**FIGURE 3:**
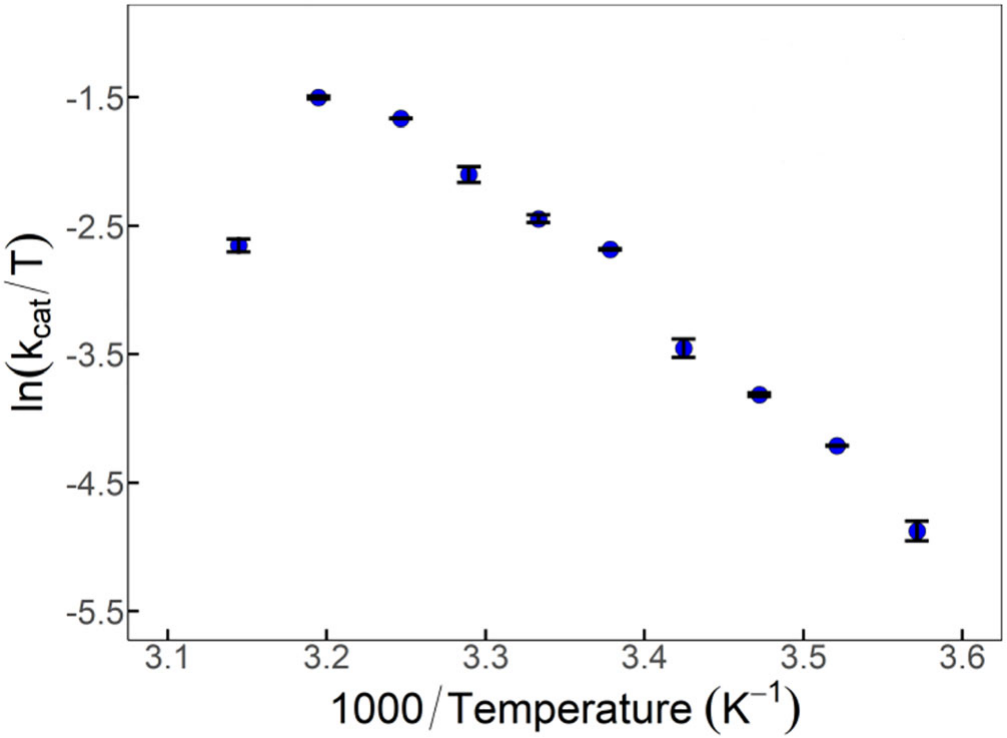
Eyring plot of *Pn*PEPCK. The Eyring plot shows the temperature activity relationship of *Pn*PEPCK (blue) for the PEP carboxylation reaction; data were measured in duplicate and error bars (standard errors) may be hidden within data point.

Subsequently, Eyring plots for *Pn*PEPCK were collected with finer temperature increments (Figure 3 and Table S1). Activation parameters can be derived from the slope and *y*‐intercept of Arrhenius or Eyring plots denoted here as ∆*H* ^rxn^ and ∆*S* ^rxn^ for historical purposes. When extracted from data of temperature‐dependent systems, these derived parameters have unknown thermodynamic value (McLeod et al., 2025). *Pn*PEPCK had a ∆*H* ^rxn^ and ∆*S* ^rxn^ of 18.2 ± 0.871 kcal/mol and 0.009 ± 0.005 kcal/mol•T, respectively (up to *T*_opt_ before slope inversion). We have previously published Eyring plots for rcPEPCK and the ∆*H* ^rxn^ and ∆*S* ^rxn^ values (up to 60°C) were found to be 18.7 ± 1.02 kcal/mol and 0.010 ± 0.023 kcal/mol•T (McLeod et al., 2025). Therefore, the temperature dependence of *k*_cat_ prior to *T*_opt_ is statistically indistinguishable between *Pn*‐ and rcPEPCK.

### 3.2 Global overview of cold‐adapted PEPCK complexes

*Pn*PEPCK crystallized in three unique crystal forms when the same mother liquor is supplemented with various ligands. The data quality of all structures is very high, spanning high resolution limits of 1.53–2.00 Å with near full completeness. Besides a few residues at the N‐terminus, all residues are modeled and there is very strong electron density to support modeling of added ligands. Analysis of these crystals found that there was an open holo form (P2_1_2_1_2_1_), an open bound (C222_1_), and a bound form which could be observed in either the open or closed conformation (P2_1_2_1_2_1_). The closed state in the P2_1_2_1_2_1_ open/closed crystal had a unit cell B length ~ 13 Å larger than the open state (Tables S3 and S4). Unlike rcPEPCK, none of these crystals were found to permit soaking with substrates/ligands and therefore do not appear to be able to undergo the open‐closed transition *in crystallo*. All three ternary complexes (βSP‐GTP, oxalate‐GTP, and PGA‐GDP) in addition to βSP alone were found in the closed conformation. Conversely, the holo structure and those with PEP alone were in the open conformation. PEP was found to have varying occupancy in each crystal form. PEP occupancy was 100% in the P2_1_2_1_2_1_ crystal, but only 74% (with correspondingly weaker and less continuous electron density) in the C222_1_ crystal. When nucleotide was absent, the enzyme adopted the open complexes and the P‐loop (which binds the nucleotide) was modeled in two conformations. However, a lack of strong density suggested that the loop was sampling other states (Figure S4). Residues 600–603 were somewhat disordered in the fully bound P2_1_2_1_2_1_ crystals but were well ordered in the partially occupied C222_1_ crystals despite PEP adopting an identical pose.

Comparing the various closed and open structures of *Pn*PEPCK revealed that there were very few differences between either the three open structures or the various closed structures (Figure S5 and Table S2). As expected, comparing open and closed *Pn*PEPCK complexes demonstrated residue displacements throughout the structure reflecting global and local rearrangement after ligand binding. As expected, comparing the βSP (closed) to βSP‐GTP (closed) complexes revealed significant remodeling of the nucleotide‐binding site despite both complexes adopting the closed conformation (Figure S6).

### 3.3 Comparisons between cold‐ and warm‐adapted PEPCKs

Both rc‐ and *Pn*PEPCK are GTP‐dependent PEPCKs but are from Eukaryote and Bacteria lineages, respectively. Consequently, there are significant sequence differences present throughout the structure (61.3% sequence similarity, 47.5% sequence identity). Most of these sequence changes are situated in the periphery of the enzyme scaffold (Figure 1c). After alignment, two regions on the enzymes are significantly different regardless of which complexes are compared. rcPEPCK has an extended loop, 25 residues in length (93–119), which is 8 residues shorter in *Pn*PEPCK (91–109) despite both enzymes having the same overall sequence length (622). The second region is an ordered loop in rcPEPCK (390–397) that forms a short α‐helix in *Pn*PEPCK (384–394) (Figure S7).

**FIGURE 4:**
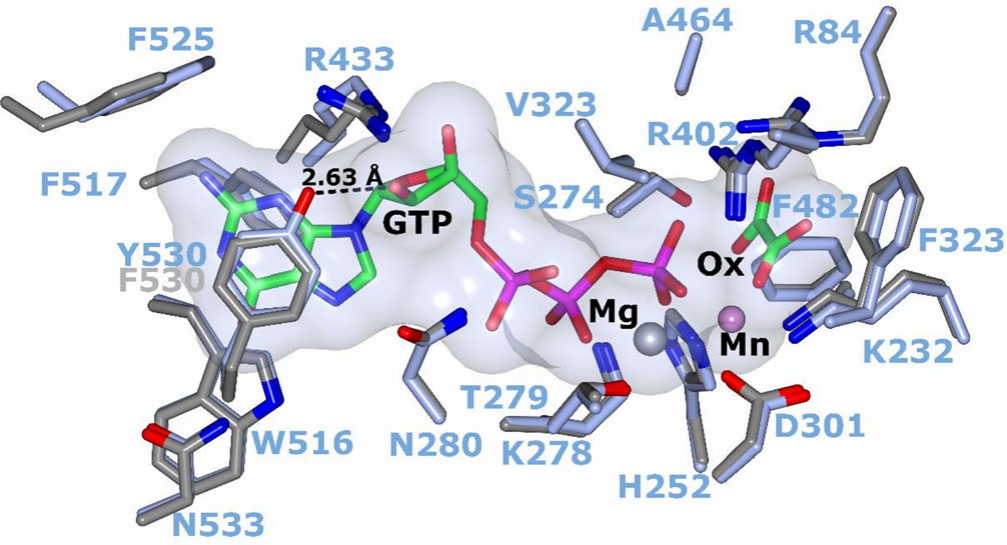
Active site conservation of psychrophilic and mesophilic PEPCKs. Alignment of rcPEPCK (PDB 3DT2—gray) and *Pn*PEPCK (ice blue), both in complex with oxalate and GTP. The active site is completely conserved except for the F530 (rcPEPCK) to Y530 (*Pn*PEPCK) substitution. The tyrosine hydroxyl of rcPEPCK forms a 2.63 Å H‐bond with O2′ of the nucleotide.

In the active site, the residues that directly coordinate with the metal cofactors, nucleotide, or substrates are nearly conserved with only a single change in the nucleotide pocket. Tyrosine 530 (*Pn*PEPCK) is substituted for phenylalanine in rcPEPCK, which results in the loss of an H‐bond between the O1 of Y530 and the ribose O2′ of GTP (Figure 4). There were slight displacements of many of the residues, with the most noticeable shift found in the nucleotide pocket. The loop containing F525 was displaced ~2 Å away from the nucleotide, which may be a result of different ground state structures between rc‐ and *Pn*PEPCK or could be due to different crystal conditions/packing artifacts. However, there was no change in the observed location of the bound nucleotide.

When comparing the active sites of the open holo‐structures of psychro‐ and mesophilic PEPCK (PDB 2QEW (Sullivan & Holyoak, 2007)), as previously mentioned, the P‐loop is somewhat disordered in *Pn*PEPCK but does exhibit enough electron density to support the modeling of two lowly populated but discrete conformations (Figure S4). In contrast, rcPEPCK is found in a singular conformation. In both enzymes, the R‐loops are well‐modeled, and the Ω‐loops are disordered in the open state. A comparison of the warm and cold‐adapted ternary complexes revealed few structural differences. rcPEPCK complexes with βSP‐GTP (PDB 3DT7 (Sullivan & Holyoak, 2008)) or PGA‐GDP (PDB 3DTB (Sullivan & Holyoak, 2008)) had two molecules in the asymmetric unit (ASU), one closed and one open, whereas the equivalent *Pn*PEPCK complexes had one closed molecule.

While there is no equivalent rcPEPCK structure for the βSP*‐Pn*PEPCK complex, there is data of the OAA‐rcPEPCK complex (1 molecule in the ASU—PDB 2QF1 (Sullivan & Holyoak, 2007)) which βSP*‐Pn*PEPCK is meant to mimic. The most significant difference between these two structures is that the βSP*‐Pn*PEPCK complex is closed, whereas the OAA‐rcPEPCK complex is open (1.24 Å global Cα RMSD). The OAA‐rcPEPCK complex indicates that OAA is modeled in two conformations, each with partial occupancy. One is present in the substrate (OAA/PEP) binding pocket, while the other OAA wraps around the M1 metal, forming a chelated metal complex that bridges both OAA/PEP and nucleotide binding sites (Sullivan & Holyoak, 2007). The P‐loop is also disordered in this complex. For *Pn*PEPCK, βSP does not appear to populate the bridging position, but it aligns well with the other OAA/PEP binding site pose (Figure S8). Additionally, the P‐loops are now in a single well‐modeled conformation in the βSP*‐Pn*PEPCK complex.

Comparing PEP*‐Pn*PEPCK and PGA‐rcPEPCK (2 molecules in the ASU—PDB 2RKA (Stiffin et al., 2008)) complexes (1.02 Å global Cα RMSD) revealed that PEP binds in a similar pose as one of the two modeled conformers in the PGA‐rcPEPCK complex (Figure S8). Here, the P‐loop was mostly disordered and modeled as two conformations in the PEP*‐Pn*PEPCK P2_1_2_1_2_1_ complex, partially ordered and modeled as two conformers in the PEP*‐Pn*PEPCK C222_1_ complex, and was ordered in the PGA‐rcPEPCK complex.

### 3.4 Evolutionary changes to Ω‐loop closure mechanism

**FIGURE 5:**
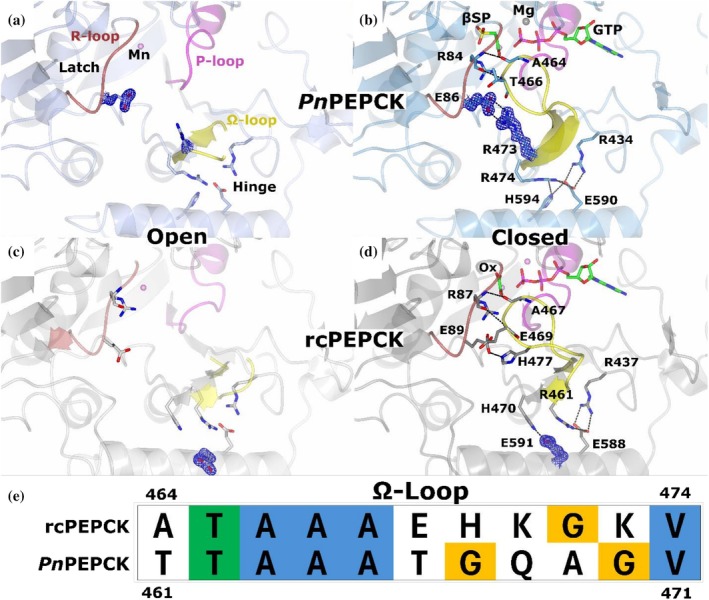
Active site changes during the transition between open and closed states of psychrophilic and mesophilic PEPCK. Active sites of PEPCKs undergo conformational changes as they transition from open (unbound or PEP bound) to closed (bound) states. (a) Holo (P2_1_2_1_2_1_) *Pn*PEPCK (ice blue). (b) βSP‐GTP *Pn*PEPCK complex (closed) (light blue). (c) rcPEPCK holo (PDB 2QEW) (open) (gray) active site. (d) rcPEPCK bound with oxalate and GTP (PDB 3DT2) (closed) (dark gray). Active site loops and atoms are colored as described above (Figure 1); in addition, magnesium—gray, manganese—pink. 2*F*_o_ − *F*_c_ electron density maps are rendered at 1.7*σ*. (e) Alignment of the Ω‐loop for both PEPCKS. Letters are colored by ClustalΩ annotation.

For PEPCK to operate correctly, the Ω‐loop must achieve a delicate equilibrium between disordered (open) and ordered (closed) conformations. This equilibrium is sensitive to active site ligation, as has been illustrated in structure–function and mutagenesis studies of rcPEPCK (Cui et al., 2017; Johnson et al., 2016; Johnson & Holyoak, 2010; Johnson & Holyoak, 2012). It has been demonstrated that two structural elements are critical to this dynamic transitioning: the “latch” and the “hinge” (Figures 1b and 5a). Examination of the structures of *Pn*PEPCK illustrated the presence of similar latch and hinge elements present in the cold‐adapted enzyme. In the open conformation of *Pn*PEPCK, both the Ω‐loop and R473 of the hinge region are disordered (Figure 5a). After closure and Ω‐loop remodeling, R473 formed a bidentate salt bridge with E86 (R‐loop), stabilizing the Ω‐loop over the active site. The rest of the hinge region of *Pn*PEPCK (R474, R434, H594, and E590) was unchanged between open and closed transitions. Once closed, the latch (apex) was observed to form two backbone interactions between R84 (R‐loop) and both T466 and A464 (Figure 5b). In contrast, when rcPEPCK is open, E591 of the hinge region is rotated away from its binding partner H470 (Figure 5c), and upon closure, E591 rotates towards H470, forming its salt‐bridge interaction. The latch of rcPEPCK is stabilized by a salt bridge between H477 and E89 (R‐loop) and the two aforementioned conserved backbone interactions between R87 (R‐loop) and both E469 and A467 (Figure 5d) (Sullivan & Holyoak, 2008).

## 4 DISCUSSION

The thermal adaptation of enzymes has often been studied through the lens of thermostability, with little emphasis on the evolved differences in the structure–function relationship. Many studies have categorized enzyme‐specific intramolecular interactions, focused on flexibility, and used few (typically apo state) crystal structures that only capture a narrow swath of conformational states associated with activity. To glean insight into the mechanisms of psychrophilic enzyme activity, we have collected temperature‐dependent kinetic measurements and a comprehensive set of structural measurements of complexes that represent many states sampled during substrate capture (*k*_cat_/*K*_M_) and turnover (*k*_cat_) of an enzyme from a cold‐adapted organism. We compared these to prior complementary measurements of a well‐characterized mesophilic variant.

At first glance, the kinetic data suggest that *Pn*PEPCK does not have all “classical” traits associated with psychrophilicity; however, psychrophilic characteristics are becoming less stringent as more diverse enzymes are studied and trends are evaluated by examining all published data compared to a per‐system basis (Feller, 2003; Lockwood & Somero, 2012; Miller, 2017; Somero, 1995; Stark et al., 2022). Classical psychrophilic traits include low melting temperatures (*T*_M_), increased *K*_M_ values (Dong et al., 2018), higher activity at room‐temperature (Georlette et al., 2003; Hobbs et al., 2012), a less temperature‐dependent *k*_cat_, and a reduced optimal temperature (*T*_opt_) when compared to meso‐ and thermophilic enzymes (Feller, 2003; Fields & Somero, 1998; Georlette et al., 2003; Georlette et al., 2004; Somero, 1995). A recent meta‐analysis suggests that some of these characteristics may be due to a small sample size and may not be universally conserved psychrophilic properties (Stark et al., 2022). This meta‐analysis indicated that neither *k*_cat_ nor *K*_M_ (or *k*_cat_/*K*_M_) at room‐temperature correlate with *T*_growth_. However, *T*_*M*_ does positively correlate with *T*_growth_ (Stark et al., 2022). There also does not appear to be any significant difference in the temperature dependency of *k*_cat_ with thermophilicity when looking at all available data (Elias et al., 2014). Even the more general idea of a stability–activity tradeoff has been called into question (D'Amico et al., 2003; Lonhienne et al., 2000; Miller, 2017). The result of this incongruency is that it remains unclear if there are strict rules of thermal adaptation of activity and highlights the need for more comprehensive and specific correlations to be established between the structure and function of differentially adapted thermal variants.

Both rc‐ and *Pn*PEPCK exhibit similar thermal stability (Figure 2) with a surprisingly slightly greater thermostability for *Pn*PEPCK when assessed by checking activity after incubation at various temperatures. In contrast, when thermal melting curves were collected by differential scanning fluorimetry, *Pn*PEPCK had lower thermostability compared to rcPEPCK for each complex tested. The magnitude of the difference in thermostability diminished after binding with GTP or GTP and oxalate (Table 1). The discrepancy between the two experiments may be due to the fact that thermal unfolding is both a temperature‐ and time‐dependent phenomenon, and the time required to collect data with each approach is different. What is most interesting is the significant instability of *Pn*PEPCK in the unliganded state and the stabilization afforded in the case of both enzymes (reduction in the difference in *T*_M_ values between the pair) upon binding of GTP. It seems plausible that the general observation that psychrophilic enzymes are less thermostable than meso‐ or thermophilic enzymes is valid, but the difference may be overstated. In the future, determination of thermostability should be evaluated with and without ligands to account for the ligand‐induced *T*_M_ shifts. Finally, the *T*_M_ data does indicate that *Pn*PEPCK was folded at *T*_opt_ and the activity inversion is not due to denaturation. We note that while these measurements were made under similar conditions to the other kinetic measurements presented here, the thermostability will likely differ from *in vivo* as the influences of the cellular milieu such as protein–protein interactions (chaperones or by crowding), or salt/lipid interactions could further (de)stabilize the enzyme folds (Fusco et al., 2022).

For the PEP ➔ OAA reaction, the temperature dependence of *k*_cat_ for *Pn*PEPCK is statistically identical to that of rcPEPCK (Figure 3), suggesting that the temperature dependence of the underlying events controlling the rate‐determining step(s) is likely similar between the two enzymes. Our recent multi‐temperature crystallography study on rcPEPCK suggests that the temperature dependence of *k*_cat_ likely occurs due to the physicochemical properties of the active site, where both the enzyme and ligands were found to increasingly populate active conformations as temperature increased (McLeod et al., 2025). The observed changes were hypothesized to increase the likelihood of phosphoryl transfer, which was previously established to be at least partially rate‐limiting for *k*_cat_ at room temperature (Johnson et al., 2016). The conservation of both the active site residues and the structure of the closed conformation further supports the notion that the temperature dependence of *k*_cat_ is a local effect originating from the active site that influences phosphoryl transfer in both PEPCKs (Figure 4).

*Pn*PEPCK does exhibit both a raised *K*_M_ (and decreased *k*_cat_/*K*_M_) and a steeper temperature dependence of *K*_M_ compared to rcPEPCK for the PEP carboxylation reaction (Table 2). Thus, at all temperatures measured, *Pn*PEPCK has a decreased probability of capturing substrate and committing it to turnover compared to rcPEPCK. *Pn*PEPCK also has a lowered *T*_opt_ (40°C) compared to rcPEPCK (60°C) (Figure 3). The Eyring plot shape shows that immediately after *T*_opt_ the enzyme is still active, and further increasing temperature decreases activity. This suggests that as temperature increases beyond *T*_opt_, some unique process in *Pn*PEPCK, such as a change in the rate‐determining step, leads to an abrupt decrease in the likelihood of turnover. Both the increased *K*_M_ and lowered *T*_opt_ are traditional characteristics of psychrophilicity (Feller, 2003; Siddiqui & Cavicchioli, 2006).

To elucidate the structural origins of the differences between *k*_cat_/*K*_M_ and *T*_opt_ for the PEP carboxylation reaction in mesophilic and psychrophilic PEPCK, we collected crystal structures of the various complexes representing points on the reaction coordinate. These structures included the holo form, enzyme bound with the substrate/substrate analogs (PEP, βSP), as well as substrate analog‐nucleotide complexes (βSP‐GTP, oxalate‐GTP, or PGA‐GDP). We note that peripheral sequence and structural changes like extended/truncated or altered structures of loops make it difficult to correlate the changes of distal elements with the observed kinetic effects. We therefore focus our analysis on the active site elements that have a well‐characterized structure–function relationship in rcPEPCK. Of the active site residues directly coordinating the substrates/ligands, there is only a single Y–F (warm–cold) substitution that is present in the nucleoside binding cleft which likely does not contribute significantly to catalysis and likely affects nucleotide binding more (Figure 4). There are also few changes to the Ω‐loop which may provide an avenue to the observed psychrophilic kinetic properties (Figure 5E). These observations are consistent with a recent survey of sequences from thermally adapted enzymes coupled with biochemical analysis of ketosteroid isomerase that has suggested that few residue changes are required to achieve thermal adaptation (Pinney et al., 2021).

**FIGURE 6:**
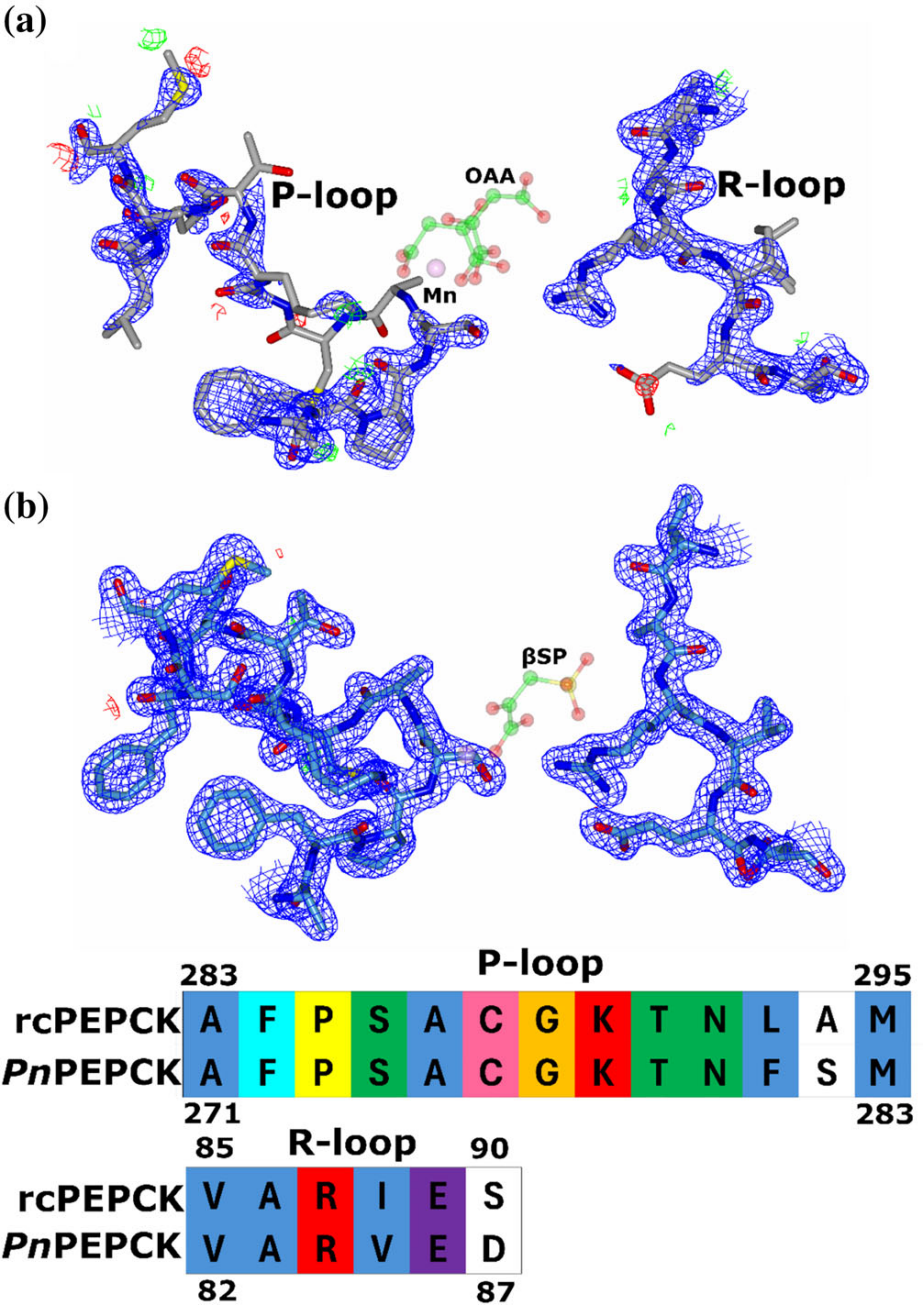
Decreased P‐loop disorder after βSP binding may contribute to preferential closure of *Pn*PEPCK. (a) The OAA‐rcPEPCK complex indicates that the P‐loop is still relatively disordered which may clash with Ω‐loop closure. (b) The βSP‐*Pn*PEPCK complex indicates the P‐loop is ordered and in the quasi‐closed conformation, resulting in Ω‐loop closure. 2*F*_o_ − *F*_c_ (1.7*σ*—blue) and *F*_o_ − *F*_c_ (3.0*σ*—green/red) electron density maps are shown. Below are the sequence alignments of the P‐ and R‐loops.

PEPCK's catalytic function is strongly dependent on the necessity for PEPCK's Ω‐loop to undergo a disorder‐to‐order transition, despite the structural element itself not being used in the chemical transformation directly (Cui et al., 2017; Johnson et al., 2016; Johnson & Holyoak, 2010; Johnson & Holyoak, 2012). More specifically, the Ω‐loop must be disordered (open) for substrates to bind and must then become ordered (closed) for the reaction to proceed. Premature opening leads to pyruvate formation by solvent protonation of the enol–pyruvate intermediate (Johnson & Holyoak, 2010; Johnson & Holyoak, 2012). Finally, the lid must reopen to release the products and restart the catalytic cycle. Based upon these mechanistic principles, we suggest that an evolutionary pressure such as temperature could alter the sequence of the Ω‐loop and surrounding regions that play a role in its dynamic behavior to conserve loop function in different environments. These alterations could impact loop dynamics such that elevated temperatures could impair the activity of the psychrophilic enzyme.

For rcPEPCK, closure has only been observed after the formation of the ternary complex, suggesting that the binding energy of both substrate and nucleotide is required to induce closure. *Pn*PEPCK on the other hand, appears to be able to close without nucleotide present if βSP occupies the substrate binding pocket. Structural and mutagenesis data support a mechanism where rcPEPCK closure is induced after the nucleotide binds the P‐loop, and the substrates bind the R‐loop, and both loops undergo a conformational change to a more ordered conformation. Concomitant with loop ordering are global changes that bring the active site loops towards the reaction center, allowing the Ω‐loop to close and form the latch with the R‐loop. When just the nucleotide is bound (PDB 2QEY) to the P‐loop, the R‐loop is disordered and cannot form the latch with the Ω‐loop. When just OAA (PDB 2QF1) or oxalate (PDB 2RK7) binds the R‐loop, the P‐loop is disordered and/or in an open conformation which prevents Ω‐loop closure (Figure 6a). When *Pn*PEPCK is in the holo state or when PEP is bound, the P‐loop is disordered (modeled as two states—Figure S4) and the R‐loop is well ordered in the closed conformation; fulfilling one of two requirements for closure and resulting in *Pn*PEPCK and the Ω‐loop being open. When βSP is in complex with *Pn*PEPCK, the P‐loop adopts a quasi‐closed conformation (quasi‐closed because when nucleotide binds, the P‐loop's nucleobase cleft undergoes a conformational change to the fully closed state—Figure S6) (Figure 6b). The P‐loop, now pinned in this quasi‐closed conformation, fulfills both active site loop requirements and removes the aforementioned steric clash so that the Ω‐loop can close. It is unclear why there is this difference between the variants, whether it is due to sequence changes in the periphery changing the free‐energy landscape (as the R‐ and P‐loop are nearly conserved—Figure 6) or if it is due to βSP ligand itself not recapitulating all aspects of the OAA complex it is supposed to mimic. The consequence that this complex can induce closure more readily than the mesophilic variant may result in decreased substrate capture of GTP in the OAA ➔ PEP reaction as premature closure would prevent the formation of the Michaelis complex and thus turnover. This propensity for closure after OAA/βSP binding is likely inconsequential in the Michaelis–Menten parameters for measured PEP ➔ OAA reaction as this would represent a substrate‐release step and is not known to be rate‐limiting for turnover but the possibility cannot be ruled out.

As the latch and hinge of the Ω‐loop are directly contributing to the closure mechanism, these two regions may be responsible for the varying temperature dependence of *k*_cat_/*K*_M_ and *T*_opt_. rcPEPCK adopts the closed conformation by a conformation change that results in E591 rotating towards an already established network of residues (R461, E588, and R437) to interact with H470 while the latch forms three interactions with the R‐loop; most notably E89 forming a salt bridge with H477 (Figure 5c,d) (Cui et al., 2017). *Pn*PEPCK appears to take a similar but distinct approach where the established network is fixed (R434, R474, E590, and H594) and the hinge change occurs by R473 reaching towards the latch region to interact with E86, replacing the E89–H477 interaction (Figure 5a,b). *Pn*PEPCK's closure mechanism therefore results in the loss of one enthalpic interaction compared to rcPEPCK. In conjunction with this, the H477–R473 substitution will result in a more entropically costly closed state, as arginine has a greater number of accessible side chain conformers than histidine in its uncoordinated state. The consequence being that the increased entropic barrier from the H–R substitution may lead to a decrease in the likelihood of the formation and maintenance of the latch in *Pn*PEPCK. Sustained lid closure becomes rarer, leading to a reduction in substrate capture and commitment to turnover, resulting in the observed order of magnitude reduction in *k*_cat_/*K*_M_ when compared to rcPEPCK. At higher temperatures, the ensemble of arginine side chain conformers will broaden and since *Pn*PEPCK's closed state is stabilized by fewer enthalpic interactions, these may be insufficient to counteract this disorder to maintain closure. As a result, the residency time for the closed state for *Pn*PEPCK may decrease with temperature resulting in new dynamic rate‐determining step, catalysis becoming rare, and reducing *k*_cat_ after *T*_opt_.

Mechanisms of low‐temperature thermal adaptation likely vary depending on the evolving scaffolds needs. However, the current results allow us to posit a potential general mechanism for enzymes such as PEPCK whose catalytic function requires them to transition between a catalytically incompetent, open, disordered state to a catalytically competent, closed, ordered state. In these cases, substitutions towards more intrinsically dynamic residues in cold‐adapted enzymes such as the arginine substitution illustrated here allows for balanced dynamics at cold temperatures. At low temperatures, reduced *T*Δ*S* narrows the conformational ensemble. By nature of the lower temperature, cold‐adapted enzymes would require fewer stabilizing interactions to maintain the competent conformation. This mechanism is supported here by the observation that *Pn*PEPCK utilizes fewer interactions after lid closure compared to the mesophilic rcPEPCK. However, to avoid becoming “trapped” in the ordered, closed conformation at low temperatures, the fewer enthalpic interactions are tempered with substitutions which increase the lids entropy. Taken together, this balance created by fewer, but more dynamic, residues allows the enzyme to cycle through catalysis at the reduced temperature. This hypothesis can be evaluated by comparing functional dynamics of more thermal‐adapted enzyme pairs and by creating chimeric enzymes where warm‐adapted motifs are transplanted into cold‐adapted scaffolds to control for these interactions. In addition to future experimental work, this collection high‐resolution psychrophilic and mesophilic PEPCK structural data, high quality kinetic data, and well‐described reaction coordinate detailing structural and chemical mechanism of activity sets PEPCK as suitable model system for study with molecular dynamics simulations in order to quantize these highlighted differences both temporally (such as lid residency times) and energetically (the temperature dependence of interactions). These experimental findings could aid in constraining simulations and conclusions by providing a relatively unbiased ground‐truth.

While there has been significant emphasis on uncovering general design strategies that enhance thermostability (Eijsink et al., 2004; Eijsink et al., 2005; Howell et al., 2014), these changes often result in enzymes that do not retain optimal or thermophilic functionality (Jing et al., 2017). Mechanistic studies evaluating structural changes that lead to thermally adapted functionality are fewer, but as done here, could offer insight into specific ways in which we can engineer enzymes with a given goal in mind such as increased turnover at low or high temperature. The implications are obvious when attempting to design thermally adapted enzymes for bioprocessing/manufacturing, but insights such as these can also be important in *de novo* enzyme design with new or optimized functionality.

## AUTHOR CONTRIBUTIONS

**Matthew J. McLeod:** Conceptualization; investigation; validation; formal analysis; writing – original draft; writing – review and editing; data curation; methodology. **Shauhin Yazdani:** Investigation; formal analysis. **Sarah A. E. Barwell:** Investigation. **Todd Holyoak:** Conceptualization; writing – review and editing; formal analysis; supervision; funding acquisition; resources; project administration.

## FUNDING INFORMATION

TH acknowledges funding support from the Natural Science and Engineering Research Council (NSERC) of Canada.

## Supporting information


**FIGURE S1:** DSF derivative plots for rcPEPCK in the absence and presence of ligands. Thermal denaturation profiles are shown for rcPEPCK alone (violet), rcPEPCK + oxalate (green), rcPEPCK + GTP (orange), and rcPEPCK + GTP + oxalate (blue). Vertical dashed lines indicate melting temperatures (*T*_M_) derived from peak minima: 52.0, 54.4, 61.4, and 64.7°C, respectively. Ligand binding increases protein stability, with the largest shift observed for the GTP + oxalate combination. All samples contained 1 mM MnCl_2_.
**FIGURE S2:** DSF derivative plots for *Pn*PEPCK in the absence and presence of ligands. Denaturation profiles are shown for *Pn*PEPCK alone (violet), *Pn*PEPCK + oxalate (green), *Pn*PEPCK + GTP (orange), and *Pn*PEPCK + GTP + oxalate (blue). Vertical dashed lines mark melting temperatures (*T*_M_) calculated from peak minima: 42.7, 46.3, 59.5, and 61.7°C, respectively. Initial denaturation is observed in *Pn*PEPCK alone. Ligand binding increased protein stability, with GTP and oxalate together producing the greatest *T*_M_ shift relative to *Pn*PEPCK. All samples contained 1 mM MnCl_2_.
**FIGURE S3:** The temperature dependency of *k*_cat_/*K*_M_. The catalytic efficiency of rcPEPCK (gray) and *Pn*PEPCK (blue) are shown for the PEP carboxylation reaction. Raw data is present in Table 1.
**FIGURE S4:** PEPCKs P‐loop in the holo state. (a and c) *Pn*PEPCK's P‐loop is modeled in two conformations (pink and teal) but electron density is still relatively sparse. (b and d) rcPEPCKs P‐loop is well ordered (PDB 2QEW). 2*F*_o_ − *F*_c_ electron density map is rendered at 1.0 *σ* for (a) and (b), and 1.5 σ for (c) and (d) (blue). *F*_o_ − *F*_c_ map is rendered at 3 *σ* in all panels (green/red).
**FIGURE S5:** Cα RMSD differences per residue rc‐ versus *Pn*PEPCK. RMSD difference between open rc‐ and *Pn*PEPCK complexes (holo—blue), or closed rc‐ and *Pn*PEPCK complexes (βSP‐GTP complex, orange). There are several regions of large RMSD deviations that are primarily surface loops or changes in secondary structure. The chain average RMSD deviation is 0.737 and 0.728 Å for the open and closed complexes respectively.
**FIGURE S6:** Conformational changes to nucleotide binding site between *Pn*PEPCK βSP and βSP‐GTP complexes. βSP (pale blue) and βSP‐GTP (dark blue) complexes were superimposed to show conformational changes in both backbone and side chain positioning after nucleotide binding. Atoms are colored by type where carbons are shown in green (ligand) or chain color (residues).
**FIGURE S7:** Loop extension and secondary structure changes. rcPEPCK (gray) and *Pn*PEPCK (cornflower blue) βSP‐GTP complexes alignment indicate two regions with significant structural changes. *Pn*PEPCK residues 385–393 form a short alpha‐helix (top—cyan) while rcPEPCK is a short loop (black). Residues 100–103 (bottom—cyan) are truncated compared to rcPEPCK (black). Active site R‐loop (red), P‐loop (magenta), Ω‐loop (yellow) and M1, M2, nucleotide, βSP are shown and colored by type.
**FIGURE S8:** Ligand pose changes between *Pn*‐ and rcPEPCK. (a) Binary complexes of βSP (OAA—2QF1) and (b) PEP‐P2_1_2_1_2_1_ (PGA – 2RKA) for *Pn*PEPCK (rcPEPCK) indicate generally conserved binding positions, albeit the rcPEPCK structures indicate that there are secondary competing binding positions. *Pn*PEPCK is colored in ice blue, rcPEPCK in gray. Atoms are colored by type.
**TABLE S1:** Kinetic values used in Figure 3—Eyring plot for *Pn*PEPCK.
**TABLE S2:** Average global Cα RMSD (Å) and conformational state of each complex.
**TABLE S3:** Crystallographic statistics for polarPEPCK complexes (open).
**TABLE S4:** Crystallographic statistics for polarPEPCK complexes (closed).

## Data Availability

The data that support the findings of this study are available from the corresponding author upon reasonable request.
