# Supplementary material for: Structural mechanisms for cold‐adapted activity of phosphoenolpyruvate carboxykinase
Source: Protein Sci. 2025 Oct 16;34(11):e70326. doi: 10.1002/pro.70326 (PMC12529880; doi:10.1002/pro.70326)
Supplement: Supplementary file 1 — FIGURE S1: DSF derivative plots for rcPEPCK in the absence and presence of ligands. Thermal denaturation profiles are shown for rcPEPCK alone (violet), rcPEPCK + oxalate (green), rcPEPCK + GTP (orange), and rcPEPCK + GTP + oxalate (blue). Vertical dashed lines indicate melting temperatures (T M) derived from peak minima: 52.0, 54.4, 61.4, and 64.7°C, respectively. Ligand binding increases protein stability, with the largest shift observed for the GTP + oxalate combination. All samples contained 1 mM MnCl2. FIGURE S2: DSF derivative plots for PnPEPCK in the absence and presence of ligands. Denaturation profiles are shown for PnPEPCK alone (violet), PnPEPCK + oxalate (green), PnPEPCK + GTP (orange), and PnPEPCK + GTP + oxalate (blue). Vertical dashed lines mark melting temperatures (T M) calculated from peak minima: 42.7, 46.3, 59.5, and 61.7°C, respectively. Initial denaturation is observed in PnPEPCK alone. Ligand binding increased protein stability, with GTP and oxalate together producing the greatest T M shift relative to PnPEPCK. All samples contained 1 mM MnCl2. FIGURE S3: The temperature dependency of k cat/K M. The catalytic efficiency of rcPEPCK (gray) and PnPEPCK (blue) are shown for the PEP carboxylation reaction. Raw data is present in Table 1. FIGURE S4: PEPCKs P‐loop in the holo state. (a and c) PnPEPCK's P‐loop is modeled in two conformations (pink and teal) but electron density is still relatively sparse. (b and d) rcPEPCKs P‐loop is well ordered (PDB 2QEW). 2F o − F c electron density map is rendered at 1.0 σ for (a) and (b), and 1.5 σ for (c) and (d) (blue). F o − F c map is rendered at 3 σ in all panels (green/red). FIGURE S5: Cα RMSD differences per residue rc‐ versus PnPEPCK. RMSD difference between open rc‐ and PnPEPCK complexes (holo—blue), or closed rc‐ and PnPEPCK complexes (βSP‐GTP complex, orange). There are several regions of large RMSD deviations that are primarily surface loops or changes in secondary structure. The chain average RMSD dev [file PRO-34-e70326-s001.docx]

Structural mechanisms for cold-adapted activity of phosphoenolpyruvate carboxykinase

**Authors:** Matthew J. McLeod^1,2*^, Shauhin Yazdani^2^, Sarah A. E. Barwell^2^, Todd Holyoak^2*^

**Affiliations:**

^1^University of Cincinnati, Cincinnati Ohio, USA. Department of Chemistry. – Current address

^2^University of Waterloo, Waterloo Ontario, Canada. Department of Biology.

*Corresponding authors. Email: mcleodmw@ucmail.uc.edu & tholyoak@uwaterloo.ca


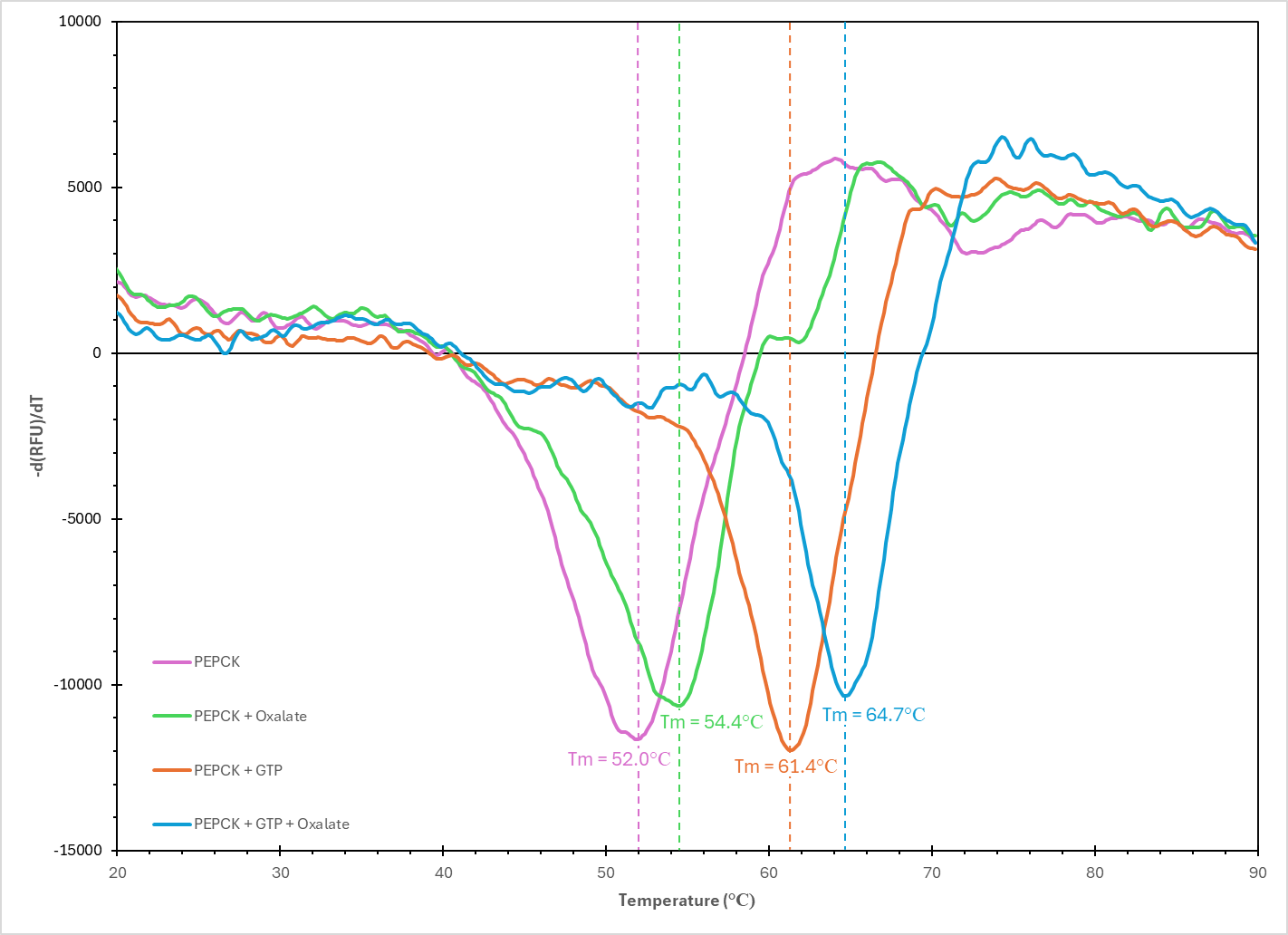


**Figure S1**: **DSF derivative plots for rcPEPCK in the absence and presence of ligands.** Thermal denaturation profiles are shown for rcPEPCK alone (violet), rcPEPCK + oxalate (green), rcPEPCK + GTP (orange), and rcPEPCK + GTP + oxalate (blue). Vertical dashed lines indicate melting temperatures (T_M_) derived from peak minima: 52.0°C, 54.4°C, 61.4°C, and 64.7°C, respectively. Ligand binding increases protein stability, with the largest shift observed for the GTP + oxalate combination. All samples contained 1mM MnCl_2_.


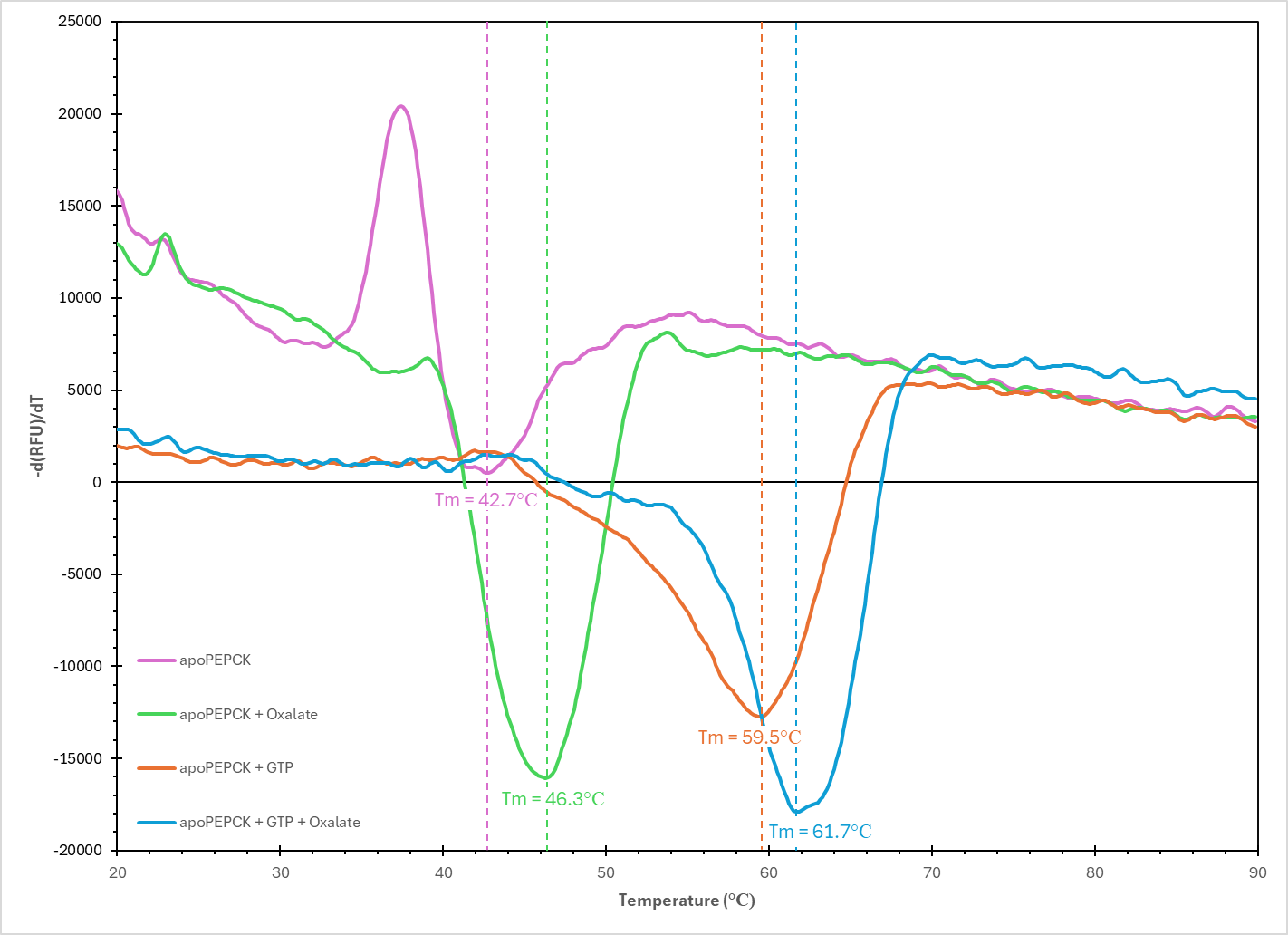


**Figure S2**: **DSF derivative plots for *Pn*PEPCK in the absence and presence of ligands.** Denaturation profiles are shown for *Pn*PEPCK alone (violet), *Pn*PEPCK + oxalate (green), *Pn*PEPCK + GTP (orange), and *Pn*PEPCK + GTP + oxalate (blue). Vertical dashed lines mark melting temperatures (T_M_) calculated from peak minima: 42.7 °C, 46.3 °C, 59.5 °C, and 61.7 °C, respectively. Initial denaturation is observed in *Pn*PEPCK alone. Ligand binding increased protein stability, with GTP and oxalate together producing the greatest T_M_ shift relative to *Pn*PEPCK. All samples contained 1mM MnCl_2_.

**Figure S3: The temperature dependency of *k_cat_*/K_M_.** The catalytic efficiency of rcPEPCK (grey) and *Pn*PEPCK (blue) are shown for the PEP carboxylation reaction. Raw data is present in Table 1.

**
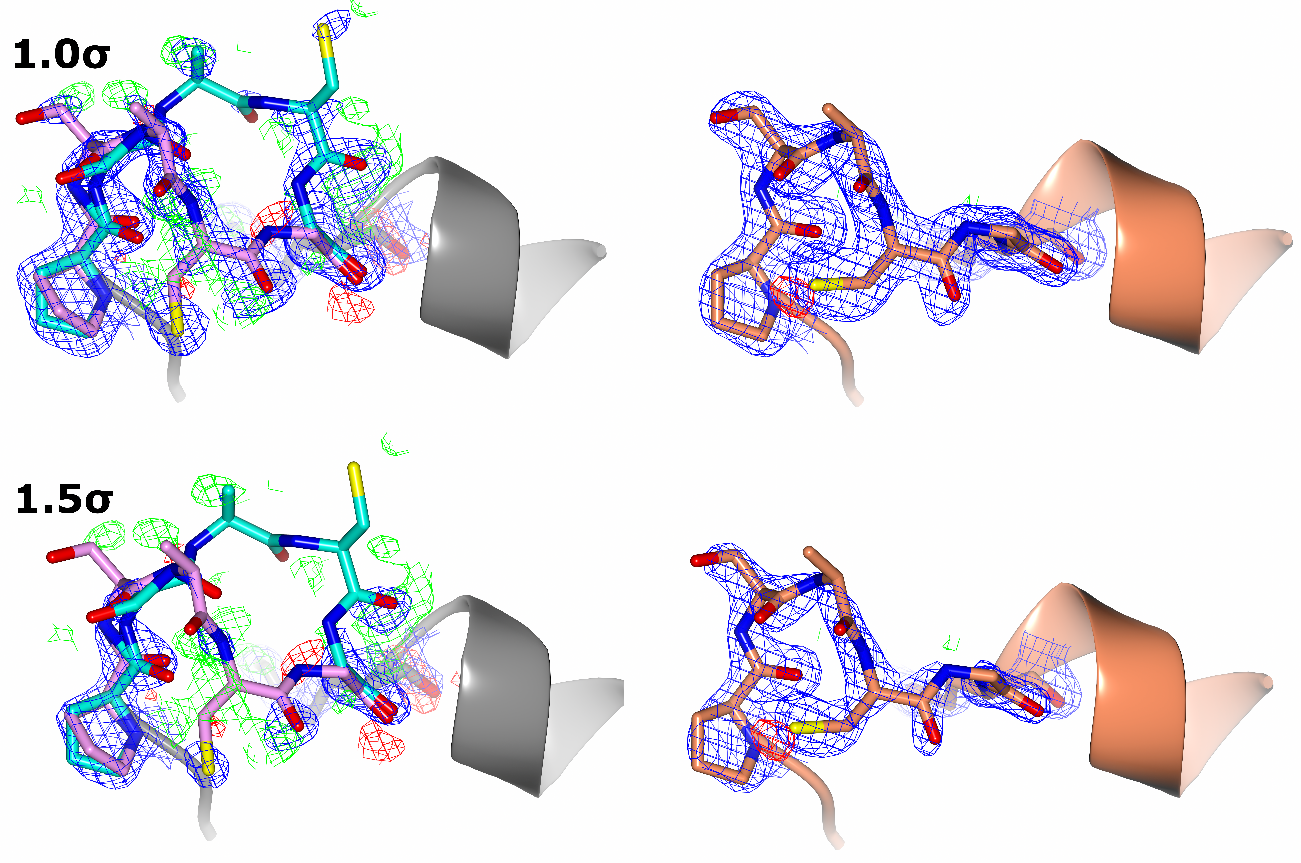
**

**D**

**C**

**A**

**B**

**Figure S4: PEPCKs P-loop in the holo state. A** and **C)** *Pn*PEPCK’s P-loop is modelled in two conformations (pink and teal) but electron density is still relatively sparse. **B** and **D)** rcPEPCKs P-loop is well ordered (PDB 2QEW). 2F_o_-F_c_ electron density map is rendered at 1.0σ for **A** and **B**, and 1.5σ for **C** and **D** (blue). F_o_-F_c_ map is rendered at 3σ in all panels (green/red).


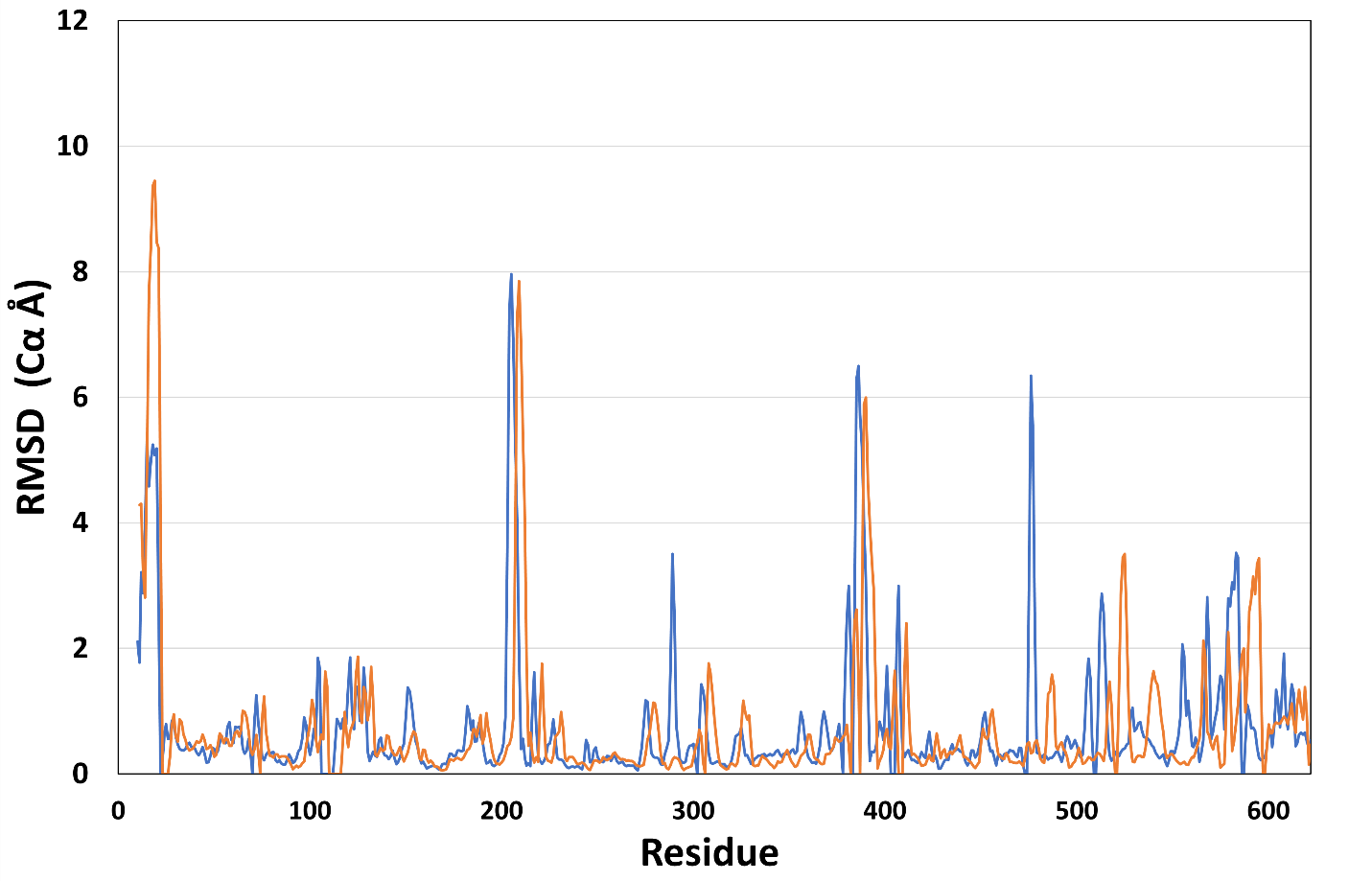


**Figure S5: Cα RMSD differences per residue rc- vs *Pn*PEPCK.** RMSD difference between open rc- and *Pn*PEPCK complexes (holo - blue), or closed rc- and *Pn*PEPCK complexes (βSP-GTP complex, orange). There are several regions of large RMSD deviations that are primarily surface loops or changes in secondary structure. The chain average RMSD deviation is 0.737 Å and 0.728 Å for the open and closed complexes respectively.


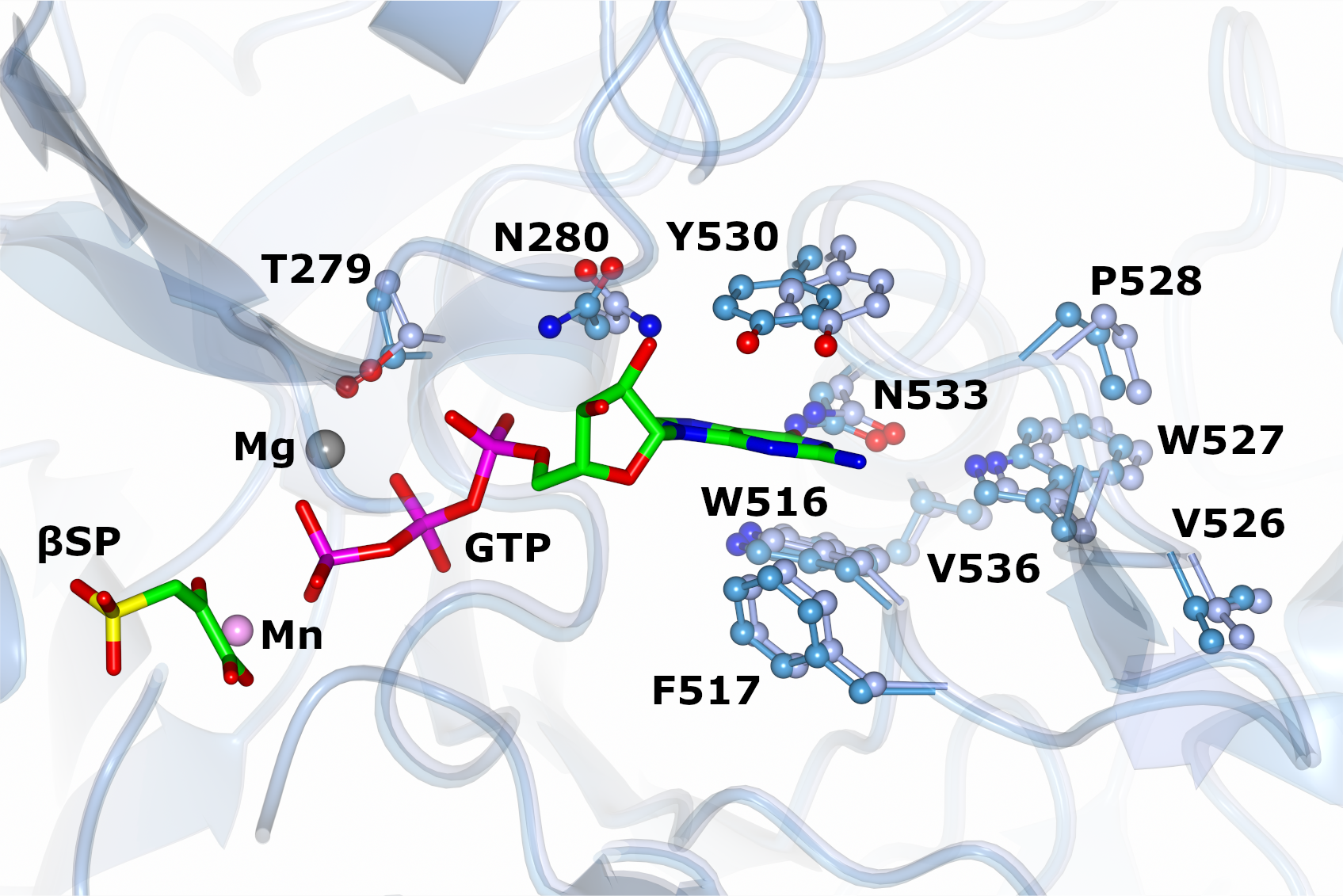


**Figure S6: Conformational changes to nucleotide binding site between *Pn*PEPCK βSP and βSP-GTP complexes.** βSP (pale blue) and βSP-GTP (dark blue) complexes were superimposed to show conformational changes in both backbone and side chain positioning after nucleotide binding. Atoms are colored by type where carbons are shown in green (ligand) or chain color (residues).


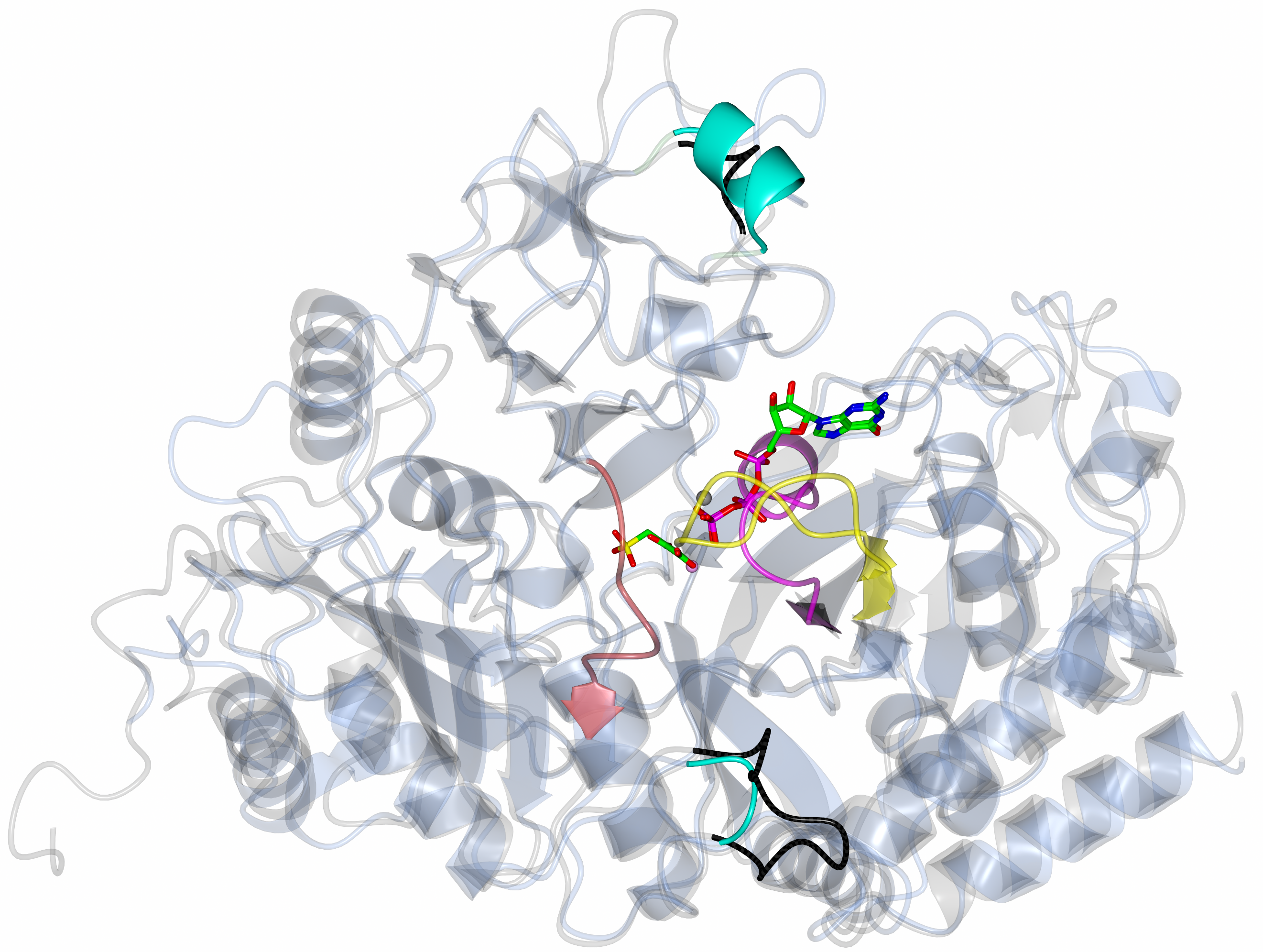


**Figure S7: Loop extension and secondary structure changes.** rcPEPCK (grey) and *Pn*PEPCK (cornflower blue) βSP-GTP complexes alignment indicate two regions with significant structural changes. *Pn*PEPCK residues 385-393 form a short alpha-helix (top – cyan) while rcPEPCK is a short loop (black). Residues 100-103 (bottom – cyan) are truncated compared to rcPEPCK (black). Active site R-loop (red), P-loop (magenta), Ω-loop (yellow) and M1, M2, nucleotide, βSP are shown and colored by type.


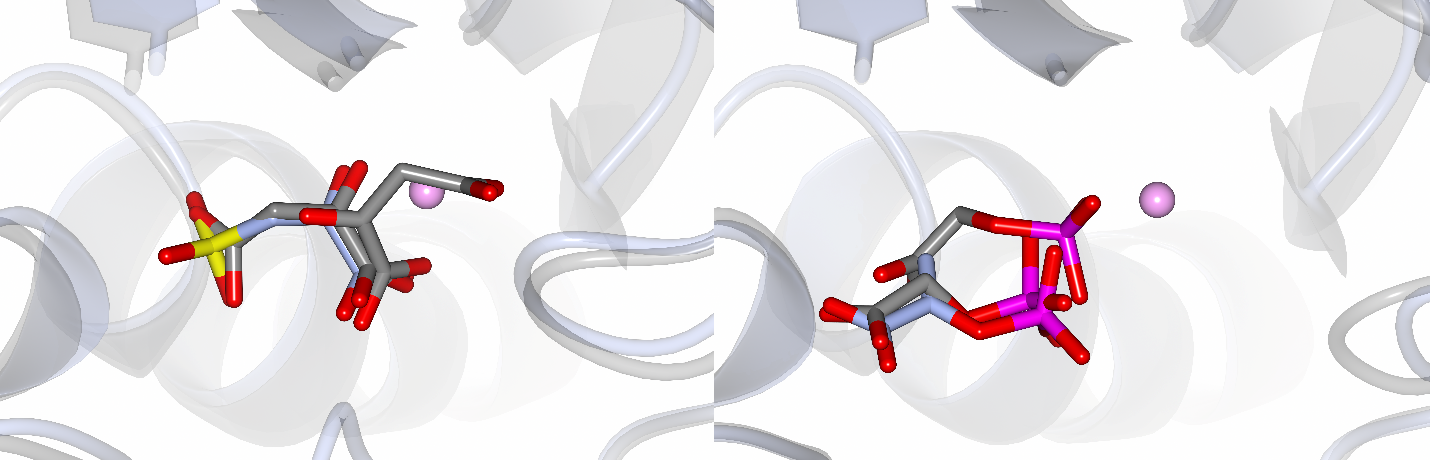


**B**

**A**

**Figure S8: Ligand pose changes between *Pn*- and rcPEPCK.** A) Binary complexes of βSP (OAA – 2QF1) and B) PEP-P2_1_2_1_2_1_ (PGA – 2RKA) for *Pn*PEPCK (rcPEPCK) indicate generally conserved binding positions, albeit the rcPEPCK structures indicate that there are secondary competing binding positions. *Pn*PEPCK is colored in ice blue, rcPEPCK in grey. Atoms are colored by type.

| **1000/T ( K)** | **ln(*k_cat_*/T)** | **ln(*k_cat_*/T)** |
| --- | --- | --- |
| 3.57 | -4.88 | -4.72 |
| 3.52 | -4.21 | -4.21 |
| 3.47 | -3.81 | -3.84 |
| 3.42 | -3.45 | -3.31 |
| 3.38 | -2.68 | -2.69 |
| 3.33 | -2.44 | -2.39 |
| 3.29 | -2.10 | -1.98 |
| 3.25 | -1.67 | -1.66 |
| 3.19 | -1.50 | -1.52 |
| 3.14 | -2.65 | -2.55 |

**Table S1: Kinetic values used in Figure 3 - Eyring plot for *Pn*PEPCK**

| **Table S2: Average global Cα RMSD (Å) and conformational state of each complex** | | | |
| --- | --- | --- | --- |
| **GDP+PGA**  **vs.**  **GTP+βSP** | **GTP+OX**  **vs.**  **GDP+PGA** | **GTP+OX**  **vs.**  **GTP+βSP** | **GTP+βSP**  **vs.**  **βSP** |
| 0.083 | 0.063 | 0.084 | 0.190 |
|  |  |  |  |
| **Holo (P2_1_2_1_2_1_)**  **vs.**  **PEP** | **Holo (P212121)**  **vs.**  **PEP (C222_1_)** | **GTP+OX**  **vs.**  **Holo (P2_1_2_1_2_1_)** | **GDP+PGA vs.**  **PEP** |
| 0.104 | 0.278 | 0.647 | 0.671 |
|  |  |  |  |
| **GTP+βSP** | **GTP+OX** | **GDP+PGA** | **βSP** |
| Closed | Closed | Closed | Closed |
|  |  |  |  |
| **PEP (P2_1_2_1_2_1_)** | **Holo (P2_1_2_1_2_1_)** | **PEP (C222_1_)** |  |
| Open | Open | Open |  |

| **Table S3: Crystallographic statistics for polarPEPCK complexes (open)** | | | |
| --- | --- | --- | --- |
|  | **Holo** | **Phosphoenolpyruvate** | **Phosphoenolpyruvate** |
| **PDB ID** | 9E32 | 9E33 | 9E34 |
| **Wavelength (Å)** | 0.9686 | 0.9686 | 0.9686 |
| **Resolution range (Å)** | 61.5 - 1.53  (1.59 - 1.53) | 81.4 - 1.74  (1.80 - 1.74) | 56.5 - 1.57  (1.63 - 1.57) |
| **Space group** | P 21 21 21 | C 2 2 21 | P 21 21 21 |
| **Unit cell (Å / °)** | 68.185 73.396 112.788 90 90 90 | 74.501 110.700 162.816 90 90 90 | 68.007 73.439 112.975 90 90 90 |
| **Total reflections** | 1067049 (54099) | 912862 (45198) | 1031698 (50690) |
| **Unique reflections** | 86713 (4275) | 69110 (3419) | 79536 (3920) |
| **Multiplicity** | 12.3 (12.7) | 13.2 (13.2) | 12.9 (12.9) |
| **Completeness (%)** | 100 (100) | 100 (100) | 100 (100) |
| **Mean I/sigma(I)** | 8.4 (0.3) | 13 (0.3) | 8.7 (0.3) |
| **Wilson B-factor (Å^2^)** | 22.4 | 31.8 | 22.11 |
| **R-merge** | 0.142 (1.758) | 0.076 (1.829) | 0.101 (1.854) |
| **R-meas** | 0.148 (1.821) | 0.079 (1.902) | 0.106 (1.931) |
| **R-pim** | 0.042 (0.505) | 0.022 (0.519) | 0.029 (0.534) |
| **CC1/2** | 0.998 (0.777) | 0.999 (0.633) | 0.999 (0.625) |
| **Reflections used in refinement** | 80761 (5488) | 69061 (6835) | 79441 (7830) |
| **Reflections used for R-free** | 3821 (251) | 3446 (347) | 4070 (362) |
| **R-work** | 0.195 (0.399) | 0.202 (0.306) | 0.178 (0.285) |
| **R-free** | 0.229 (0.411) | 0.233 (0.335) | 0.203 (0.316) |
| **CC(work)** | 0.968 (0.777) | 0.962 (0.787) | 0.966 (0.811) |
| **CC(free)** | 0.949 (0.739) | 0.941 (0.693) | 0.963 (0.775) |
| **Number of non-hydrogen atoms** | 5268 | 5120 | 5241 |
| **macromolecules** | 4747 | 4706 | 4756 |
| **ligands** | 8 | 13 | 25 |
| **solvent** | 513 | 403 | 460 |
| **Protein residues** | 605 | 603 | 607 |
| **RMS(bonds) (Å)** | 0.004 | 0.004 | 0.007 |
| **RMS(angles) (°)** | 0.7 | 0.65 | 0.93 |
| **Ramachandran favored (%)** | 97.2 | 98.2 | 96.2 |
| **Ramachandran allowed (%)** | 2.83 | 1.84 | 3.48 |
| **Ramachandran outliers (%)** | 0 | 0 | 0.33 |
| **Rotamer outliers (%)** | 1.01 | 0.41 | 0.4 |
| **Clashscore** | 3.31 | 5.93 | 3.08 |
| **Average B-factor (Å^2^)** | 25.7 | 38.0 | 25.6 |
| **macromolecules** | 24.9 | 37.6 | 24.7 |
| **ligands** | 35.9 | 38.5 | 32.2 |
| **solvent** | 32.2 | 42.6 | 33.9 |

| **Table S4: Crystallographic statistics for polarPEPCK complexes (closed)** | | | | |
| --- | --- | --- | --- | --- |
|  | **β-sulfopyruvate** | **β-sulfopyruvate - GTP** | **Oxalate – GTP** | **Phosphoglycolic acid - GDP** |
| **PDB ID** | 9E35 | 9E36 | 9E37 | 9E38 |
| **Wavelength (Å)** | 0.9686 | 1.1271 | 1.54 | 0.9686 |
| **Resolution range (Å)** | 68.6 - 1.71  (1.77 - 1.71) | 53.5 - 1.80  (1.87 - 1.80) | 30.5 - 2.00  (2.07 - 2.00) | 68.4 - 1.52  (1.58 - 1.52) |
| **Space group** | P 21 21 21 | P 21 21 21 | P 21 21 21 | P 21 21 21 |
| **Unit cell (Å / °)** | 68.785 86.097 113.423 90 90 90 | 68.523 85.6069 113.799 90 90 90 | 68.489 85.557 112.857 90 90 90 | 68.419 85.649 113.634 90 90 90 |
| **Total reflections** | 946234 (47183) | 815205 (41002) | 49489 (4379) | 1317514 (65816) |
| **Unique reflections** | 72952 (3592) | 62527 (3061) | 45093 (4114) | 101818 (4977) |
| **Multiplicity** | 13.0 (13.1) | 13.0 (13.4) | 13.1 (6.70) | 12.9 (13.2) |
| **Completeness (%)** | 100 (100) | 100 (100) | 99.2 (88.9) | 99.5 (98.7) |
| **Mean I/sigma(I)** | 6.0 (0.3) | 4.6 (1.0) | 21 (2.2) | 7.0 (0.2) |
| **Wilson B-factor (Å^2^)** | 27.6 | 16.3 | 30.6 | 26.6 |
| **R-merge** | 0.175 (2.909) | 0.280 (2.140) | 0.119 (0.637) | 0.129 (3.139) |
| **R-meas** | 0.182 (3.029) | 0.292 (2.222) | 0.124 (0.686) | 0.135 (3.262) |
| **R-pim** | 0.050 (0.836) | 0.080 (0.597) | 0.033 (0.244) | 0.037 (0.881) |
| **CC1/2** | 0.997 (0.649) | 0.988 (0.727) | 0.962 (0.824) | 0.998 (0.647) |
| **Reflections used in refinement** | 70331 (5339) | 62408 (6034) | 44974 (4114) | 90605 (4824) |
| **Reflections used for R-free** | 3464 (265) | 3114 (343) | 2143 (219) | 4477 (247) |
| **R-work** | 0.189 (0.416) | 0.159 (0.257) | 0.164 (0.254) | 0.170 (0.417) |
| **R-free** | 0.230 (0.424) | 0.192 (0.287) | 0.198 (0.307) | 0.206 (0.423) |
| **CC(work)** | 0.970 (0.788) | 0.968 (0.903) | 0.971 (0.910) | 0.975 (0.846) |
| **CC(free)** | 0.950 (0.861) | 0.965 (0.861) | 0.961 (0.892) | 0.964 (0.877) |
| **Number of non-hydrogen atoms** | 5322 | 5680 | 5384 | 5589 |
| **macromolecules** | 4820 | 4885 | 4809 | 4944 |
| **ligands** | 34 | 70 | 66 | 37 |
| **solvent** | 468 | 725 | 509 | 608 |
| **Protein residues** | 615 | 615 | 615 | 615 |
| **RMS(bonds) (Å)** | 0.007 | 0.007 | 0.002 | 0.007 |
| **RMS(angles) (°)** | 0.8 | 0.89 | 0.6 | 0.89 |
| **Ramachandran favored (%)** | 97.1 | 97.7 | 97.6 | 98.2 |
| **Ramachandran allowed (%)** | 2.94 | 2.28 | 2.45 | 1.79 |
| **Ramachandran outliers (%)** | 0 | 0 | 0 | 0 |
| **Rotamer outliers (%)** | 0.4 | 0 | 0 | 0.19 |
| **Clashscore** | 2.09 | 2.35 | 1.15 | 2.44 |
| **Average B-factor (Å^2^)** | 31.8 | 18.6 | 32.6 | 29.8 |
| **macromolecules** | 31.2 | 16.9 | 31.8 | 28.7 |
| **ligands** | 38.6 | 18.6 | 34.5 | 36.9 |
| **solvent** | 37.7 | 29.9 | 40.1 | 38.5 |
